# How seas whisper to snow: teleconnections drive spatio–temporal variability of snow cover in Western Himalayas

**DOI:** 10.1038/s41598-025-18606-6

**Published:** 2025-10-06

**Authors:** Shairik Sengupta, Rajarshi Das Bhowmik

**Affiliations:** Interdisciplinary Center for Water Research, Indian Institute of Science, Bengaluru, 560012 India

**Keywords:** Cryospheric science, Hydrology, Atmospheric dynamics, Cryospheric science, Hydrology

## Abstract

The high Himalayas host a vast cryosphere, critical for both local and global environmental stability. While a few former studies have examined point-scale precipitation variabilities; others have focused on snow cover (SC) trends at regional scales. We investigate how SC in the Western Himalayas varies across spatio-temporal scales; and what factors drive these variabilities. Towards this, we derive a historical record (1989–2025) of SC for six high-mountainous watersheds from satellite (Landsat) observations. We calculate three characteristics of SC extent: Fractional SC, fractional Temporary SC, and Principal Components (PC) of Normalized Difference Snow Index. Eigen-vectors of the PCs establish the association between the spatial distribution of SC variability and basin topography. The study found that these SC characteristics are driven substantially by the North Atlantic and The Pacific at interannual time scales. The study found that several climate variability modes drive separate SC components during different seasons. In general, during winter and summer, SC variations are primarily influenced by the North Atlantic, Arctic, and Pacific. In monsoon and fall, apart from local hydroclimatic conditions, the Indian Ocean plays as a major regulator of SC, along with the Pacific. Overall findings of the study could be crucial for season-ahead SC predictions.

**Supplementary Information:**

The online version contains supplementary material available at 10.1038/s41598-025-18606-6.

## Introduction

In the cold dry deserts of the western Himalayas, water is regarded as one of the highest offerings as water is both free and priceless. All life forms – human or endangered by humans^1–3^, residents of an environment ranging from subtropical forests to alpine meadows, depend on the water cycle driven by the Himalayan cryosphere^4–7^. Humanity’s long and intricate relationship with the Himalayan cryosphere^8^ and its rivers is reflected well in the cultures of nations^9–12^. In current times direct or indirect anthropogenic actions have occasionally been protective^13,14^ of the Himalayan cryosphere, but could be detrimental as well, resulting in hydrological extremes^15–17^, floods^18–20^, landslides, temperature rises, and other environmental disasters^21–23^. While several former studies reported that anthropogenic climate change is causing glacier melt^24–27^, the Himalayan cryosphere still remains poorly understood. Hence, developing a holistic understanding of the natural variabilities of the Himalayan cryosphere is crucial for its proper protection, water sector, and overall mitigation measures.

SC in the Himalayas changes extensively over time – largely due to seasonal and sub-seasonal variations in temperature and precipitation^28^. One of the key drivers of SC is precipitation, which has been investigated by several former studies. A host of these experimental studies have suggested large-scale oceanic and atmospheric circulations (referred to as climate variability modes) to be responsible for variations in monsoon precipitation in the high elevation of the Himalayas^29^. Working at a site at the northernmost reaches of the Indian Summer Monsoon (ISM), Srivastava et al., (2017) showed using a blend of isotopic proxies that the El Niño Southern Oscillations (ENSO) have been modulating the ISM for at least the last 5500 years^30^. Singh et al., (2009) studied tree rings in the state of Himachal Pradesh and suggested that teleconnections have existed between the Pacific Decadal Oscillation (PDO), North Atlantic Oscillation (NAO), and local precipitation for almost seven centuries^31^. Sano et al., (2013) investigated more into tree rings and retrieved three centuries of isotope (δ^18^O) records to show PDO and ENSO actually have to be in the positive phase simultaneously for warmer, drier conditions to prevail in Bhutan – India’s northeastern neighbor^32^. Girgholm et al., (2009) found the fingerprint of PDO’s influence on Himalayan weather, from about a century’s worth of dust preserved in a Tibetan ice core^33^. While proxy precipitation records point towards possible connections between local and global events, only an understanding of precipitation delivering systems can truly establish the physical mechanisms of these connections. Multiple studies have shown that the Himalayas receive precipitation mostly from two major seasonal systems – Western Disturbances^34^ and Indian Summer Monsoon^35,36^. Studies combining observations and climate modeling indicate that the Arctic Oscillation (AO), the North Atlantic Oscillation (NAO), and El Niño Southern Oscillations (ENSO) influence winter precipitation in the Himalayas through alterations to the subtropical jet^37–39^. The monsoon system is known to be modulated by the ENSO and the Indian Ocean Dipole (IOD)^40^, traces of which were reflected in an isotopic study of a Himalayan Lake sediment by Meena et al., (2022)^41^. These studies, we note are limited to a few spatial locations at best, but establish the influence of climate variability modes on precipitation amounts at high altitudes. We suggest that the influence further extends to the sub-seasonal to seasonal variation of SC extent, at a regional scale – the central theme of investigation for the current study.

While computational studies have modeled precipitation amounts at the regional scale, the spatiotemporal variability in the regional SC has been largely ignored^42–44^. Himalayan SC has traditionally only been examined for trends in extent^45–48^ and duration^49,50^. Globally however, studies on trends of decreasing (e.g. in northern hemisphere^51^, Québec^52^, the Italian Alps^53^) or increasing (e.g. in the Indus Valley^54^) SC have been complemented with studies on SC variability (e.g. for the greater Alpine region^55^, and Central Asia^56^). Moreover, SC extent has been shown to respond to remote forcings in South America^57^, North America^58^^,52^, and Europe^59^. Since climate variability modes have been shown to influence SC variability across the globe, it is worth investigating the extent of SC variability, and whether climate variability modes control the spatial extent of SC at a watershed scale in Western Himalayas.

Considering this, our objectives here are:

1. To investigate the spatiotemporal variation of SC for Western Himalayan basins, and
2. To understand the long-term and seasonal drivers of SC extent, and how local meteorological variables and climate variability influence the SC extent.

**Fig. 1:**
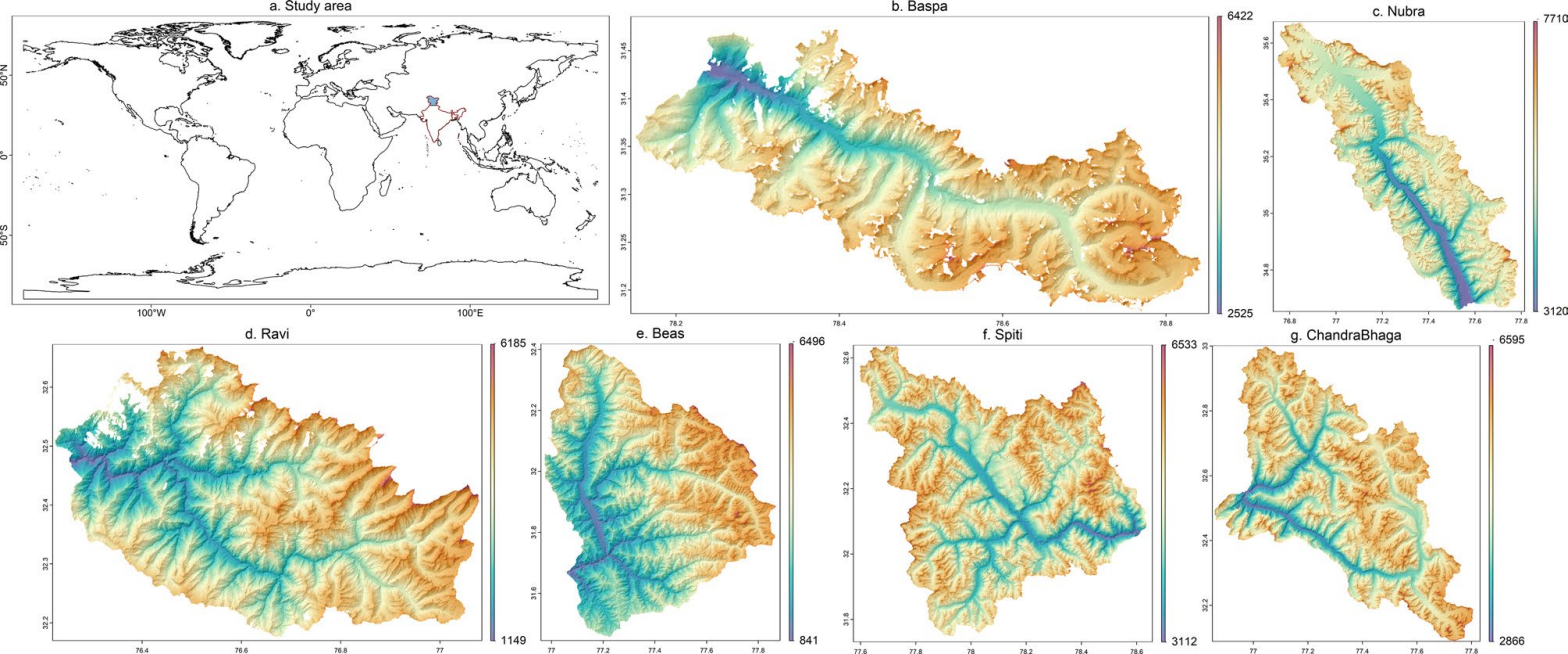
Subplot (**a**) shows the broad study area shaded in light blue, confined within the Indian borders depicted in red. Subplots (**b-g**) show the location and drainage network of the six basins in the present study, namely Baspa, Nubra, Ravi, Beas, Spiti, and ChandrwaBhaga. The legend depicts lower elevations in shades of blue and higher elevations in shades of red. The figure was prepared using R (version Race for your Life, 4.4.1) and its package terra (version 1.8–61), based on global elevation data obtained from the United States Geological Survey-Earth Explorer website.

The study addresses the broad question of the teleconnection mechanisms that enhance moisture transport^60^– whether they intensify snowfall over a larger area or facilitate an increase in snowfall over a limited region. We obtained extended Landsat images for six major river basins located In the Western Himalayas (Nubra, Spiti, Chandra-Bhaga, Ravi, Beas, Baspa, shown in Fig. 1) and estimated the SC extent by calculating the Normalized Difference Snow Index (NDSI).

Further, we propose a metric to identify SC (at a monthly scale) that does not persist through a season and thus only contributes to the seasonal variability. Furthermore, SC across an entire watershed should not be expected to behave uniformly across varied geographical features, and hence it is worthwhile investigating if separate geographical regions respond to distinct driving forces even within the same watershed. Towards this, we computed principal components (PC) of the SC (using pixels as variables) to yield the spatiotemporal variation of SC at a basin scale. Using wavelet coherence, we found SC characteristics to be influenced by the North Atlantic and The Pacific over longer time scales. Finally, through correlation and best subset regression we found several distinct oceanic atmospheric couplings to be driving discrete components of SC extent in each season. Overall, the study found that spatiotemporal variation of SC extent in western Himalayas is driven by separate teleconnection mechanisms, across different timescales.

## Results

### Long term variabilities: Uncovering snow cover components

**Fig. 2:**
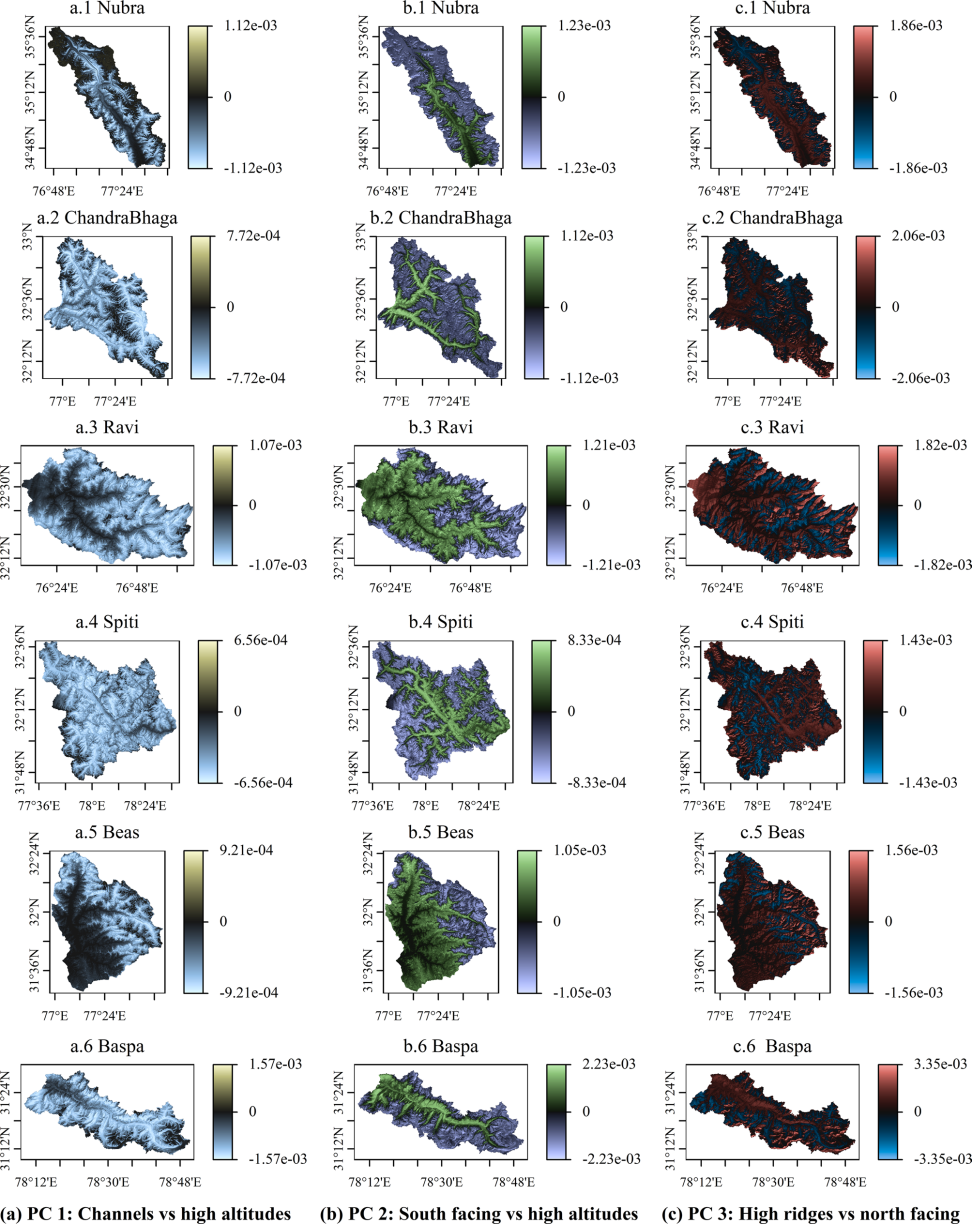
Spatial distribution of the loadings of the first three principal components (PCs), for all basins in the study area. Loadings of the first PCs are shown in the first column (subplots a.1 – a.6), bright yellow indicates a high positive while bright blues are indicative of high negative values. The loadings of the second PCs are given in the second column (subplots b1 – b6), where high positive values are depicted in bright green, and high negative values take on bright purple shades. The third column (subplots c.1 – c.6) holds the loadings of third PCs, high positive values appear as bright red, and high negative values are shown in bright blue. The figure was prepared using R (version Race for your Life, 4.4.1) and its package terra (version 1.8–61), after processing snow cover data obtained from the United States Geological Survey-Earth Explorer website.

Figure 2 presents the loadings (i.e., the eigenvectors) related to the first three principal components (PC) across six basins. The green color (blue) indicates high positive (negative) values of PC loadings. Principal components highlight contrasts between two areas, identified by higher absolute values of the loadings (variance explained by the first three PCs are given in Supplementary Table 1, and their climatologies are given in Supplementary Fig. 1). While the signs of the loadings are arbitrary, they describe opposite behaviors – in our case, the growth versus depletion of SC. Topography of the basins, i.e., the elevation, slope, and the aspect in compass direction, are given in supplementary information (Supplementary Fig. 2). The principal components obtained over the whole study period describe independent modes of inter-seasonal variations. The loadings of the first principal component (Figs. 2(a.1 – a.6)) exhibit that they capture the SC difference between high altitude areas and the river channels, as the spatial variation in the loadings coincides with the spatial variation in surface elevation (Supplementary Fig. 2(a)). This difference is lowest in monsoon, as higher temperatures and rainfall melt the accumulated snow almost completely in the basins (Supplementary Fig. 1(a)). We note that only the highest altitudes experience SC during monsoon – either by retaining SC from previous seasons, or by receiving fresh snowfall from the same weather systems delivering rain to the lower altitudes.

Further, the second principal component yields the contrast between south-facing areas and high altitudes (Figs. 2(b.1 – b.6) and Supplementary Figs. 2(a & c). Note that all six basins in the study area are well above the tropic of cancer, and hence the sun always shines from the south^61^. We found from the time series of the PCs that the contrast is highest in summer – a time of high illumination and warming due to high solar declination angles (Supplementary Fig. 1(b)). Similarly, the contrast is lower in monsoon due to SC depletion (as mentioned earlier), and in winter due to higher SC spanning the entirety of the basins. Furthermore, the third principal component highlights the difference between high ridges and north-facing regions (refer to loadings shown in Figs. 2(c.1 – c.6), and Supplementary Fig. 2). North-facing regions are cooler than the south facing regions due to the illumination effects previously described. This contrast is least pronounced during winters when the illumination differences and temperatures are lower in general, and most amplified in summers (Supplementary Fig. 1(c)). Weather systems causing cloud formations during parts of the day possibly act as a control of the third principal component. We see that the interpretations of the PCs remain the same across the six basins of the study area (Fig. 2), hinting at common underlying mechanisms controlling SC variations in all the basins – the theme of the subsequent section.

### Unveiling influences on long term variabilities

**Fig. 3:**
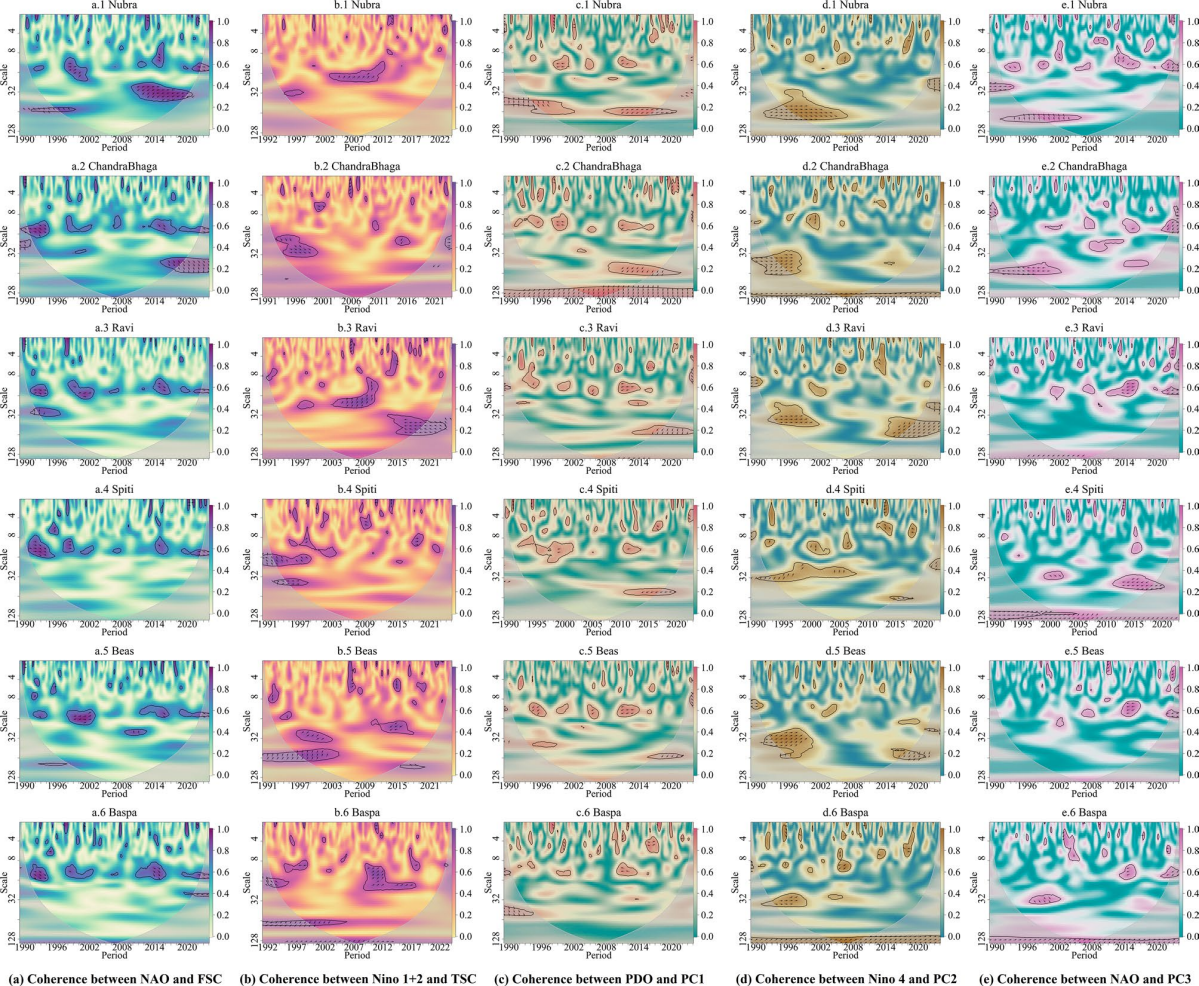
Wavelet coherence between SC parameters and oceanic-atmospheric indices. Only the drivers that act on all the basins are given. The first column (subplots a.1 – a.6) shows the relationship between FSC and NAO, purple indicates high coherence. The second column (subplots b.1 – b.6) depicts the connection between TSC and Nino 1+2. In the third column (subplots c.1 – c.6), the association between PC1 and PDO is shown. The fourth column (subplots d.1 – d.6) conveys the connection between PC2 and NINO 4. Finally, the fifth column (subplots e.1 – e.6) displays the connection between PC3 and NAO. Note that the scale axis (y) in unit of months, is not linear – each tick represents twice the time gap from the previous tick from the top. The figure was prepared using R (version Race for your Life, 4.4.1) and its package biwavelet (version 0.20.22).

Results related to the wavelet coherence analysis are presented in Fig. 3. In the current analysis, wavelet coherences between climate variability modes, and the first three PCs of NDSI, Fractional Snow Cover (FSC), and Fractional Temporary Snow Cover (TSC) were calculated. To establish Landsat derived FSC and TSC as reliable metrics against observed data, the climatologies of FSC, TSC, and observed local temperature and precipitation (details in data and methods section) have been provided in Supplementary Fig. 3. Wavelet coherence reveals potential oceanic-atmospheric controls on the behavior of SC over longer periods of time. Here, we explore patterns that are common to all the basins in the study area.

We found that the FSC is coherent with NAO at a yearly frequency. This coherence is best seen every four to six years, which is also known to be the frequency of NAO (Figs. 3(a.1 – a.6)). The TSC appears to be driven by oscillations of the southern Pacific (Nino 1+2), at a biennial frequency (Figs. 3(b.1 – b.6)). Variations in first principal component are observed to be coherent with fluctuations in the northern Pacific (i.e., PDO) at an annual frequency for about four years from 2009 to 2015 (Figs. 3(c.1 – c.6)). The second principal component is possibly influenced by the southern Pacific (Nino 4), at a four-to-five-year time scale (Figs. 3(d.1 – d.6)). Finally, changes in the third principal component are synchronized with events in the north Atlantic (NAO), at a yearly frequency (Figs. 3(e.1 – e.6)). Overall, we found that long term controls on SC likely originate in variabilities of the north Atlantic, north Pacific, and the south Pacific.

### The spatial patterns of intraseasonal variabilities

**Fig. 4:**
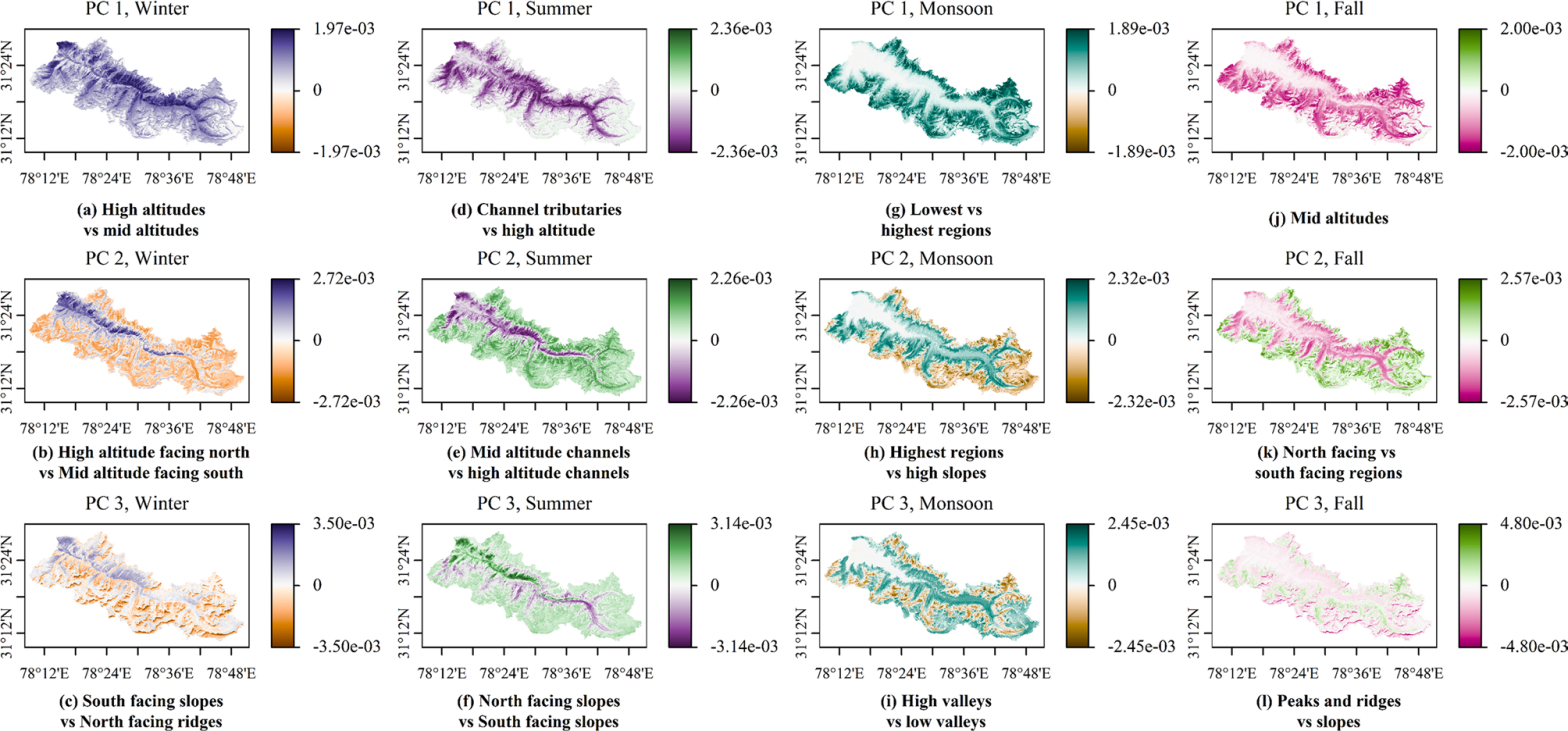
Loadings of the first three principal components of SC variations in each season for Baspa basin. Subplots a-c show loadings of PC1-PC3 of winter; high positive values appear dark blue while high negative values appear orange. The loadings of the first three PCs of summer are given in subplots d-f. Dark green represents high positive values, dark purple stands for high negative values. PC1-PC3 loadings for monsoon are displayed in subplots g-i. Once again dark green depicts high positive values, while high negative values take dark on orange shades. In subplots j-l the loadings of PC1-PC3 of fall are shown. Dark green stands for high positive values, dark pink implies high negative values. The figure was prepared using R (version Race for your Life, 4.4.1) and its package terra (version 1.8–61), after processing snow cover data obtained from the United States Geological Survey-Earth Explorer website.

The major modes of intra-seasonal variations in SC are captured in the first three principal components of each season (Winter: December, January, February; Summer: March, April, May; Monsoon: June, July, August, September; Fall: October, November). The interpretation of the PCs is similar for all the basins, so the spatial distributions of the loadings of these principal components (PC) for a representative basin, Baspa, are given in Fig. 4. Results related to the other basins are presented in Supplementary Figs. 4,5,6,7,8.

In winter, the first PC highlights the SC difference between high and intermediate altitudes (Fig. 4(a)). This difference is dominated by the latter as the major area of change in SC lies in the intermediate altitude regions. The second PC yields the contrast between high-altitude-north-facing regions and the intermediate-altitude-south-facing regions (Fig. 4(b)). This contrast results from the illumination effects described in sub-section"Long term variabilities: Uncovering snow cover components". The third PC describes the differences in SC between south-facing slopes (including the lowest elevation regions) and north-facing ridges (Fig. 4(c)). The study found that, during summer, the PC1 describes the contrast between channel tributaries and the higher altitude regions, while the variations are majorly contained in the former regions (Fig. 4(d)). This is possibly due to micro-climatic effects arising from atmospheric (in)stability and a positive feedback loop of melting snow^62^. PC2 distinguishes between mid-altitude river channels and high-altitude river channels, an effect possibly dependent on temperature fluctuations and further amplified by snow melt related feedback loops (Fig. 4(e)). The contrast captured in PC3, between north-facing and south-facing slopes (Fig. 4(f)) arises from the illumination and heating difference explained in sub-section"Long term variabilities: Uncovering snow cover components".

During monsoon, the first PC shows the contrast between the lowest and the highest altitude regions (Fig. 4(g)). This is due to not only higher temperatures during monsoon as compared to other seasons, but also higher humidity – a characteristic of the season which is particularly perfect for melting snow in widespread areas. The second PC highlights the difference between the highest regions and the high slopes (Fig. 4(h)). We note that while the higher altitudes might receive some snowfall due to altitude-driven colder temperatures capable of converting rain to snow, the valleys form a geographical bowl in which humid (and hot) conditions can prevail, creating a difference in behavior of these two regions^63^. This process can possibly be affected by extreme weather systems, which can mix up the atmosphere well. The third PC describes the contrast between high and low valleys (Fig. 4(i)). The difference between these two regions possibly arises from geographical restrictions on wind flow, combined with the altitude effect. Initial precipitation increase and subsequent decrease along an altitude gradient has been reported for winter precipitation^64^. A key takeaway here is that SC variability in monsoon appears to be entirely agnostic to illumination effects. During fall, the first PC exhibits that the major mode of variability is contained in the mid-altitudes (Fig. 4(j)). This is expected, as fall is a growing season for snow, and snow formation progresses down the altitude gradient. The second PC captures the contrast between the north-facing and the south-facing regions (Fig. 4(k)), an effect guided by illumination and affected by temperature, as explained in subsection"Long term variabilities: Uncovering snow cover components". Finally, the third PC describes the difference between the peaks and ridge regions against the slopes (Fig. 4(l)). This can arise from winter weather systems depositing snow and subsequent snow redistribution.

### Understanding the drivers of intraseasonal variabilities

**Fig. 5:**
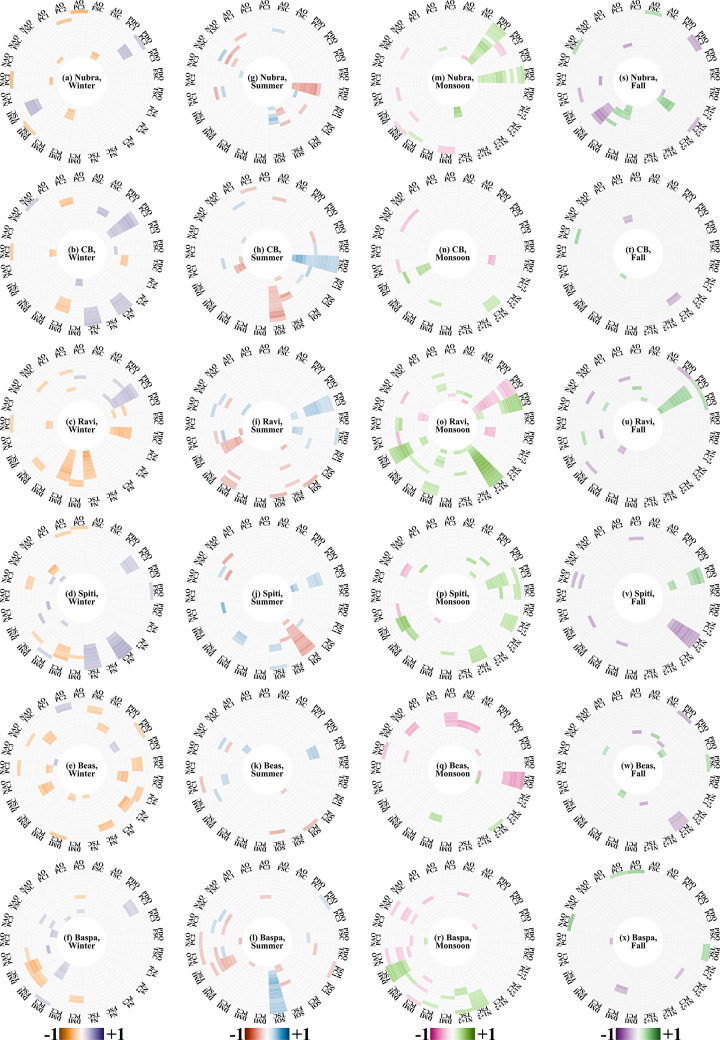
Correlation (Pearson’s r) between SC parameters (first three principal components of SC, FSC, and TSC) and potential drivers (oceanic-atmospheric indices). In all the plots, the circle closest to center represents the correlation at lag 0. Concentric circles outwards stand for increasing lags of oceanic-atmospheric indices, with the final perimeter circle standing for lag 12. Each circle is divided into 25 sectors – combinations of five oceanic-atmospheric indices and the five SC parameters that were tested for correlation. These combinations are denoted outside the perimeter. For winter (subplots a-f), the legend runs from orange to purple, denoting −1 to +1. The same range of values are represented with a red to blue legend for summer (subplots g-l). A red to green legend is used to depict the r values obtained for monsoon (subplots m-r). For fall finally (subplots s-x), r values are shown with a purple to green legend. The figure was prepared using R (version Race for your Life, 4.4.1) and its package circlize (version 0.4.16).

Figure 5 presents the results related to the correlation analysis where correlation coefficients for a basin are estimated between PCs/FSC/TSC and climate variability modes at different lags (the corresponding lower and upper bounds for the correlation coefficients are presented in Supplementary Figs. 9 & 10). Although multiple indices were considered to represent the ENSO state, eventually only the index that showed the strongest correlation with SC for a basin in each season was retained for presentation. Note that the analysis was carried out with seasonally segregated time series of predictands and predictors to address the influence of seasonality on SC. In Fig. 5, each sector is dedicated to a pair of predictor and predictand variables (say, PC1 and PDO); whereas the radial distance from the center represents the increasing lags (from 0 to 12 months) between the climate variability mode (as preceding variable) and the SC variable (as succeeding variable), and the color scale indicates the Pearson’s r coefficients (only statistically significant correlations are presented). The study did not find any noteworthy non-linear relationships between SC parameters and climate variability modes, or any common pattern across the basins thereof (Supplementary Fig. 11).

In each season, we found that a different set of climate variability modes appear as dominant drivers of SC. NAO is the most influencing control on PC2 during winter, operating on immediate lags of 0–2 months. In Spiti, this effect appears on PC1, possibly indicating a higher importance of Western disturbances in delivering snow to Spiti than in the other valleys. Another indicator of the Atlantic, AO acts at a 6–10 months lag, on PC2 for all basins except Nubra. The PC2 is also influenced by PDO with the strongest connection seen at 7–9 months lags. This effect is absent in Nubra, a high-altitude basin, Likely to be unperturbed except by the strongest outside influences. We noted that DMI, i.e., the influence of the Indian Ocean, acts on the higher and drier basins of Nubra, Chandrabhaga, and Spiti at 0–3 months lags, on PC1. In the relatively southern lower altitude basins (Ravi, Beas, and Baspa), which are more open to interactions with monsoon systems, the effects of DMI on PC2 are evident, suggesting some carry-over effect of the previous monsoon. Additionally, the valleys of Ravi and Beas are oriented such that they are the early Himalayan obstacles in the course of monsoon progression. To note, these are the only two valleys which show a stronger correlation at a higher lag of 6 months. We suspect this 6-month lag is not a teleconnection operating in winter, but rather a fingerprint of events that occurred 6 months ago from winter, in monsoon. Although Nino-4 displays minimal control over all the basins, as its influences are reflected in different characteristics of SC, no common pattern emerges. Further, in summer, NAO at lag 5 consistently emerges as a driver of PC1. However, AO does not exhibit any substantial influence on PC1. In addition, PDO at lag 5 exhibits statistically significant correlations on PC3. Notably, there are two exceptions where the PC3-PDO association is absent: Nubra and Baspa, the two basins forming the high-altitude northwestern and the low-altitude southeastern boundaries of the study area respectively. In both basins, PDO influences temporary SC instead of the more subtle PC3 at lags of 6–7 months – possibly due to the influence of stronger monsoons in these two basins. Only the strongest monsoons alter the hydroclimate in Nubra, as it lies at the ends of the monsoon’s reach. Baspa, however, is practically at the gateway of the Himalayas for monsoon winds, and Changes in monsoon activity can display a pronounced effect here. In all except two basins, we found that DMI influences the temporary SC at 0–3 months lag. The two distinct exceptions are Nubra and Baspa: Nubra shows no links between DMI and any characteristics of its summer SC, and Baspa shows a stronger association between DMI and TSC at 3–6 months lags. Finally, the influence of SOI, though varied with regard to which characteristics of SC it affects across the basins, is common in one characteristic – it is predominantly relevant at higher lags. This indicates that it plays an indirect role through its control on the summer monsoon, an effect we explore next in the subsequent season section dedicated to the monsoon season.

NAO and AO do not have a Major role to play during monsoon. The DMI appears to have an influence at a lag of 6–10 months, except in Nubra, the cold desert^65^. This suggests that the region’s teleconnection with the Indian Ocean is primarily through the monsoon system. Indeed, the other known influences on monsoon systems are also picked up by the current analysis. Effects of PDO on the SC is evident at lags of between 3 to 4 months. The effect is seen in different characteristics of SC in different basins. This is likely due to different basins interacting with the monsoon system(s) differently, due to geographical factors. Note that Nino 3.4, a well-known control of monsoons, drives various characteristics of SC at approximately 8 months lag. This apparently high lag is possibly because the ENSO states take time to develop, and it is a long chain of weather phenomena that forms the physical connection. In fall, across all basins, external influence of climate variability modes on the SC is minimal, as rapid decreases in local temperatures is evidently the major control on the SC. Local factors such as drops in the temperature and precipitation play a far more important role in SC growth as compared to the influence of climate variability modes.

The correlations between individual climate variability modes and various aspects of SC were subsequently examined through a composite analysis (results given in Supplementary Figs. 12,13,14,15,16). Composite analysis shows that the associations described previously are strengthened during periods of heightened activity of the relevant climate variability modes. Notably, the lags between NAO/AO and the SC parameters they influence reduced during heightened NAO/AO activity during winter and summer, possibly indicating a role of persistent climatic conditions over the Arctic and North Atlantic in regulating SC in the study area in these two seasons. Moreover, the effects of NAO/AO appear to be modulated through climatic conditions of wintertime Indian Ocean^66,67^. Additionally, during summers of enhanced NAO/AO activity, weather systems driven by the Arctic and North Atlantic possibly modulate the PDO driven snow delivery mechanism^68,69^. A detailed analysis is discussed in Supplementary Text 1.

**Fig. 6:**
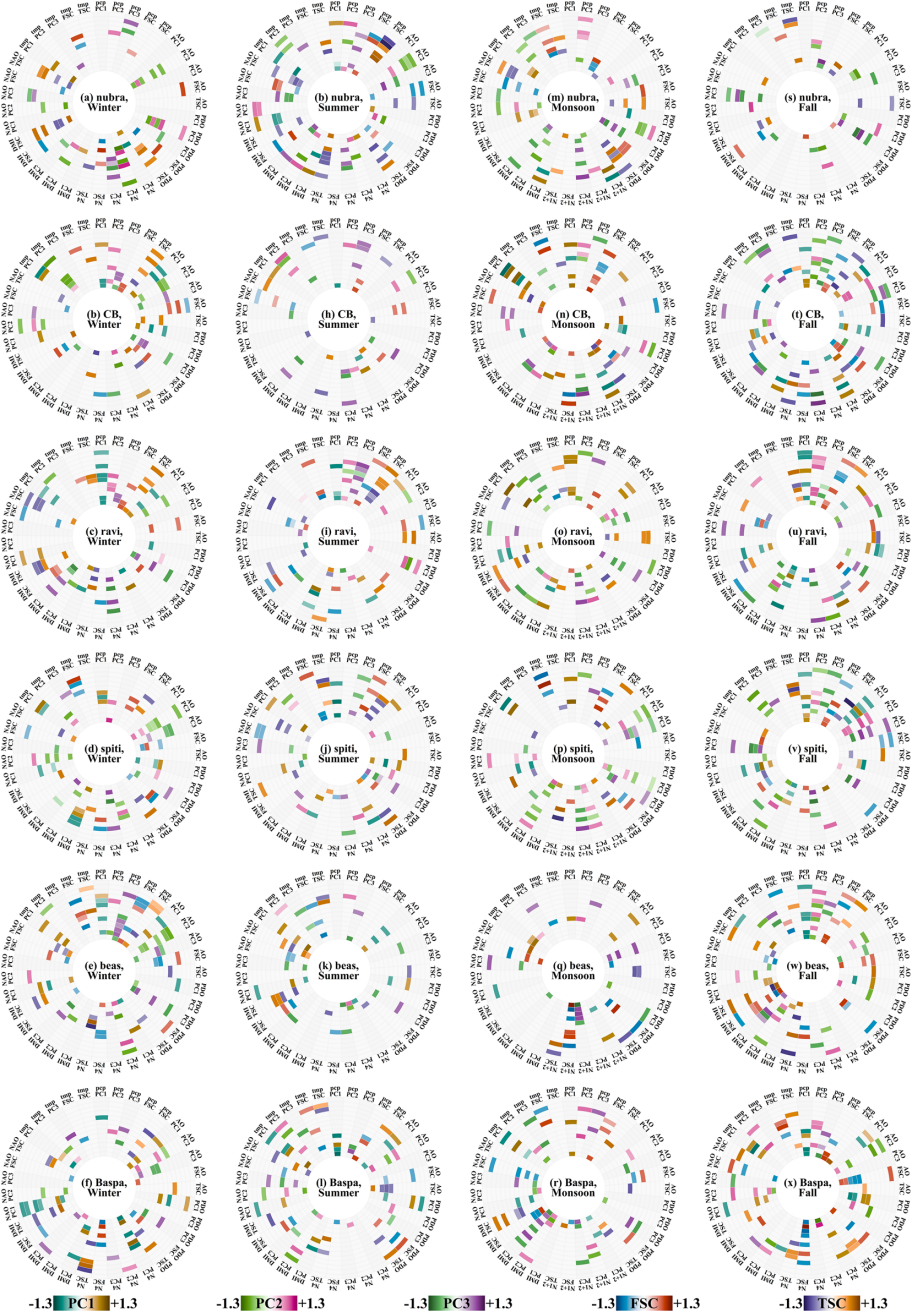
Coefficients from best subset regression. SC parameters (first three PCs, FSC, and TSC) and the independent variable (oceanic-atmospheric index, temperature and precipitation) combination is Marked on the perimeter. Concentric circles represent lags of these independent variables, with the centermost circle being lag 0 and the outermost standing for lag 12. The coefficients for PC1 are plotted in a green–brown scale, for PC2 in green-pink, and for PC3 in green-purple. The legends for FSC and TSC are in a blue-red, and orange-purple scale, respectively. The values have been plotted with a contrast stretch of the form $\frac{x}{{\left|x\right|}^{0.8}}$ to ensure that higher values do not overshadow the smaller ones. The figure was prepared using R (version Race for your Life, 4.4.1) and its package circlize (version 0.4.16).

In the current and final analysis, we attempt to answer how much influence these climate variability modes and the local drivers (precipitation and temperature) have on the SC intra-seasonal variation. Best subset regression between SC parameters and the drivers selects the optimal drivers required to develop the best linear model. Note that the study selected linear models over non-linear models since the linear correlation coefficients between SC parameters and climate variability modes were found to be statistically significant. Their relative importance, obtained in the form of regression coefficients, is presented in Fig. 6. The corresponding lower and upper bounds to the estimated regression coefficients are given in Supplementary Figs. 17 & 18, while the variance explained by each model is given in Supplementary Table 2. While the layout is similar to Fig. 5, the color scale in Fig. 6 are the coefficients of each independent variable in the best subset regression equation. Additionally, we consider the total precipitation over the basin, and spatially averaged temperature of the basin. FSC and TSC do not appear to have any particular dominating climate driver during any season. This is expected since aggregate SC characteristics like these two should actually be the resultant of an interplay between all the drivers considered. During winter (Figs. 6(a-f)), we find that the PC1 is driven primarily by NAO and PDO, with AO also having a slight contribution. The PC2 (PC3) is modulated largely by Nino 4 and PDO (AO). While in summer, conditions in the arctic and local precipitation drive PC1, PC2 is influenced largely by the southern Pacific. Intra-seasonal variations in the PC3 are in response to events in the Indian Ocean, and local precipitation. Precipitation and temperature emerge as major drivers of PC1 during monsoon. We found that PC2 and PC3 are driven by PDO, DMI, and Nino 1+2. Further, the PC3 is sensitive to AO and NAO, possibly as a fingerprint of the previous winter. In fall, although most of the drivers that showed up for the monsoon season remain active, their relative contributions vary. Precipitation emerges as a driver of all the PCs during fall. However, interestingly, temperature does not consistently come up as a driver for fall SC. This perhaps demonstrates the complicated nature of mountainous geography, where albedo effects can suddenly surpass a critical threshold and create microclimates suitable for ice formation^70,71^ (Kim et al., 2018, Wilson et al., 2020). However, microregions with low temperatures alone cannot receive snowfall, moisture in the air is also required. Hence, we found a complicated mix of drivers in the fall. Furthermore, we found that PC1, apart from precipitation, is influenced by AO and PDO, and DMI. The second and third PCs too, apart from precipitation, bear the influences of PDO and AO, respectively. Overall, we found that intra seasonal variations in SC are substantial in the six basins but varies with geomorphic characteristics of each basin, and the variation is largely influenced by a combination of climate variability modes, and local meteorological conditions.

## Discussion

**Fig. 7:**
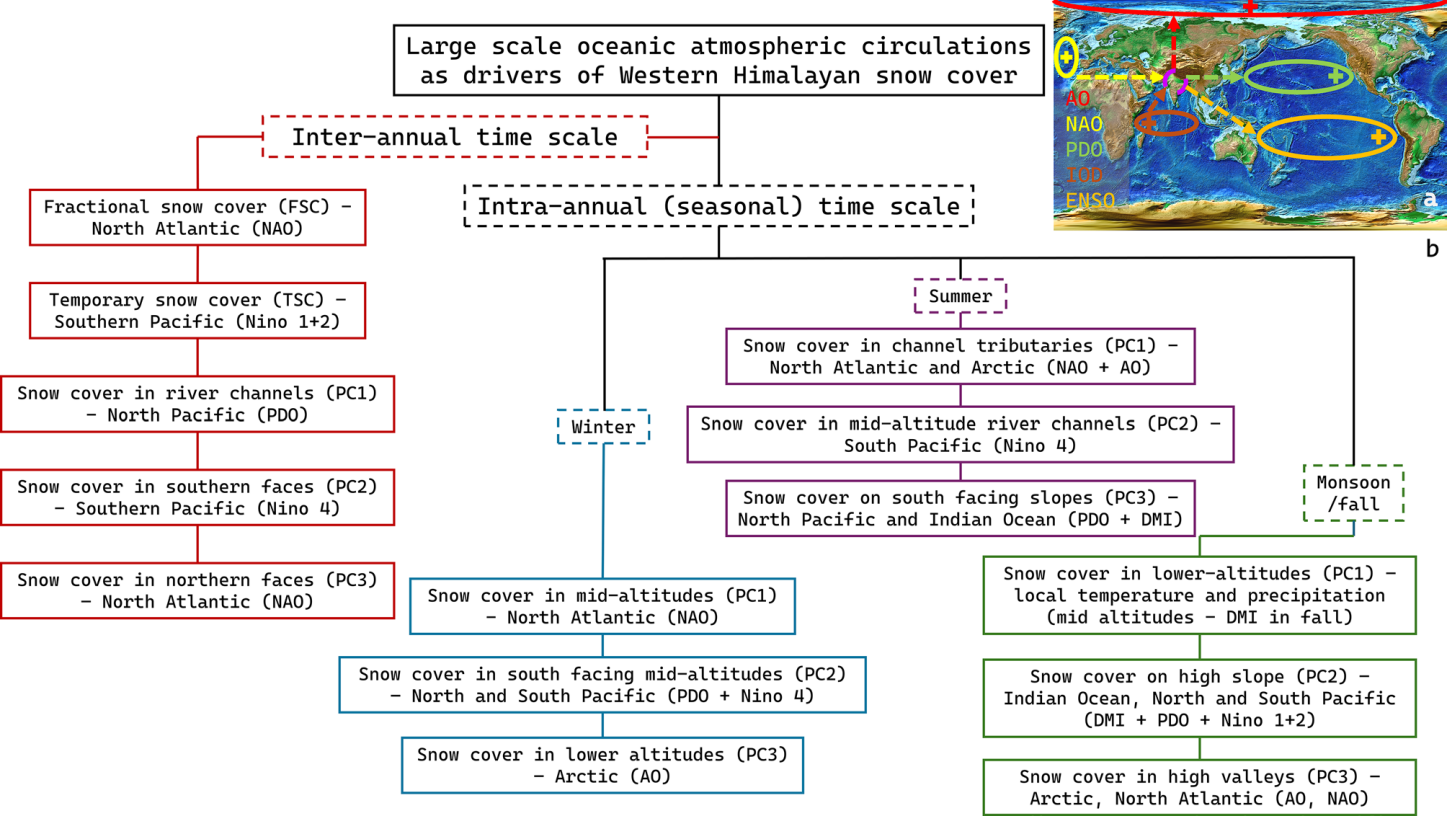
The infographic presents the climate variability modes examined in the study and their relationships with the SC characteristics observed in the study. In subplot a, AO is shown in red, NAO in yellow, IOD (DMI) in brown, PDO in green, and finally ENSO (SOI, Nino 1+2, Nino 3, Nino 3.4, Nino 4) in orange. The position of the"+"symbol indicates where warm waters are found in the positive phase of the sea surface based oceanic-atmospheric indices (IOD, ENSO, PDO). NAO and AO are not indices based on air pressure, and the "+" for these two indicate only the positive phase. Ellipses indicate roughly the region of the atmosphere or ocean described by each index. The arrows show whether moisture is transported towards (arrowhead towards India) or away from the Indian subcontinent (arrowhead away from India) during the positive phase of each climate variability mode. In subplot b, key findings from our study are summarized. The figure was prepared using Microsoft PowerPoint (version 2508).

The principal component analysis revealed that all areas covered in snow do not behave uniformly (as has been traditionally assumed) but distinct areas respond to separate local and remote factors. Large-scale ocean–atmosphere couplings are found to be driving SC extent at both longer and shorter timescales. Figure 7 summarizes the large-scale oceanic-atmospheric couplings that are found to be acting upon SC in the western Himalayas, the general direction of moisture transport when those teleconnections are active, and the SC components they influence. Prior to discussing the results from our study, we briefly present the major theories regarding the physical mechanisms of the teleconnections shown in Fig. 7(a). AO and NAO primarily influence winter precipitation India, as their positive phases lead to an increased upper-tropospheric temperature gradient, resulting in increased baroclinicity, and thus a more intense subtropical jet. This results in frequent western disturbance generation, as they grow from instabilities in the subtropical jet^72^. Moreover, AO has also been hypothesized to affect monsoon activity through a preconditioning effect on the Eurasian landmass. A negative(positive) AO results in warmer and drier (colder and wetter) conditions, which strengthens(weakens) the land-sea thermal gradient, and thus enhances(weakens) monsoon activity^73^. Conversely, a negative(positive) NAO leads to hemisphere-wide changes in wind flows and storm tracks, inducing tropospheric temperature anomalies over Eurasia. These conditions weaken(strengthen) the land-sea temperature gradient, and result in a weaker(stronger) monsoon^74^. Another climate variability mode, the IOD primarily influences monsoon precipitation, by increasing(decreasing) surface evaporation from the Indian ocean and strengthening(weakening) the monsoon system in its positive(negative) phase^75^. Further, positive IOD conditions have been shown to build negative geopotential heights over northern India, causing warm and wet southwesterly winds to be driven in from the Bay of Bengal. This translates to increased snow cover and lower temperatures in the Himalayan region that can persist through winter^76^. Furthermore, positive(negative) IOD conditions have been shown to counteract the effects of positive(negative) ENSO^77^. The ENSO system alters monsoon precipitation through alterations to both the Hadley (north–south circulation) and Walker (east–west circulation) cells^78^. The negative, La Niña phase of ENSO (positive, El Niño) strengthens(weakens) the Hadley cell, which leads to stronger(lesser) heating of the Indian ocean, resulting in increased(lesser) air mass rise and precipitation over India. On the other hand, in the Walker cell, La Niña (El Niño) causes warm waters to shift westwards (eastwards) towards Indonesia (Peru), enhancing (suppressing) upward air movement and precipitation over India. The remaining climate variability mode considered in the study – PDO – has been shown to affect atmospheric Rossby wave trains^79^. This interaction leads to positive(negative) geopotential anomalies over central Asia during cold(warm) PDO phases, resulting in a strong land-sea thermal gradient which intensifies the monsoon system^80^. Additionally, PDO in its warm(cold) phase, suppresses(enhances) the Hadley circulation over the Indo-Pacific basin, which results in upward(downward) air flow over the equatorial Indian ocean and downward(upward) air flow over the northern Indian ocean. A warmer(colder) western equatorial Indian ocean results in moisture transport away from(towards) the Bay of Bengal, resulting in weaker(stronger) monsoons^81^. Finally, PDO in its cold phase also enhances winter precipitation by increasing the heating of the tropospheric column over the north Pacific. Consequently, this anomalous heating triggers an atmospheric wave train with a trough over the Karakoram-Western Himalayas and a ridge over the Tibetan Plateau. The resulting atmospheric structure begets a stronger subtropical jet over the Western Himalayas – ultimately resulting in increased western disturbance activity^82^.

At the interannual time scale, snow cover extent (FSC) is driven by ocean-atmospheric coupling over the North Atlantic (NAO). This finding supports observational studies^34^, where western disturbances were established as a major winter precipitation source in the region. Western disturbances have been shown to be strongly associated with events in the North Atlantic^83^ and the Arctic^84^ that control the Subtropical Westerly Jet and cyclonic activities^85^. Our study found that western disturbances not only modulate snowfall amounts, but also enhance the SC extent. Additionally, the hydroclimatic state of the South Pacific (Nino 1+2) governs whether the SC persists throughout whole seasons (measured as TSC). The principal components highlight the spatial variability at the interannual time scale. The amount of SC that the river channels receive (relative to higher altitudes, represented as PC1) was found to be modulated by the North Pacific. In contrast, the south pacific appears to drive the amount of SC south facing regions receive or possibly retain (relative to higher altitudes captured as PC2). We note that the two SC characteristics that are most likely to be influenced by summer/monsoon duration and intensity are indeed affected by ENSO activity, possibly due to its modulation of heat dissipation through upper troposphere interactions^86^. A word of caution related to the wavelet analysis is warranted – while the study found significant wavelet coherence at a longer timescale, an annual coherence signature can come from seasonal cycles. As only an annual coherence is found between PC3 and NAO, we refrain from propounding any explanation for this result.

Former studies on the seasonal to interannual variation of SSTs^87,88^ align with the major finding of current study: PDO acts as a major control on SC extent on both longer and shorter time scales. The control is likely through persistent and reemerging SST patterns^89^ and subsequent forcings on the monsoon from Walker and Hadley cells^90^. The extent of SC the southern faces receive and retain (once more, relative to higher altitudes, described by PC2) is found to be driven by the ENSO cycle (Nino 4). This is due to the control exhibited by ENSO on monsoon activities, as has been explored in depth by various researchers (though their focus was primarily on rainfall) on intraseasonal^91^), interannual, and interdecadal timescales^92^ through alterations to the walker cell, especially the North African Asian Jet^93^. Monsoon activity (or lack of it) is best fingerprinted on southern faces resulting from the cloudy/clear weather during active and break phases of the season^94^. This also explains why a solar illumination-based mode of variability yields coherence with ENSO. Moreover, SC in northern faces (as before, relative to higher altitudes, picked up by PC3), is driven by climate states of the North Atlantic (NAO), a known influence on winter weather^95^.

At seasonal time scales, the correlation analysis and best subset regression reveal the influence of oceanic-atmospheric couplings on the SC in the Himalayas. During winter, we found that SC in mid altitudes (PC1) is influenced by the North Atlantic (NAO), as NAO is known to play a key role in boreal winter weather. Both the North and South Pacific (PDO and Nino 4) control SC in the south-facing-mid-altitudes (PC2), possibly through their control on the mid-altitude storms^96^. In the lowest altitudes (PC3 results), where SC may not form every winter, we found the ‘far-reaching’ consequences of the oscillations of the Arctic Vortex (AO). In summer, the major mode of SC variability is seen in the channel tributaries (PC1), which is influenced by hydroclimatic events at the North Atlantic and the Arctic (NAO and AO). This might seem odd at first, given that a predominantly winter phenomenon appears to have considerable influence in summer. This, however, is subjective to the definition of summer, as the months considered as summer in this study are just the tail end of winter in the higher latitudes. SC changes in the mid-altitude river channels (PC2 results) are driven by the Southern Pacific (SOI), due to its control on global temperatures^97^. We found the mid-altitudes to be the dominant zone of SC variations in winter. The river channels in the mid-altitudes opening up in summer might be due to valley-contained feedback loops, which are triggered by temperature changes driven by the South Pacific. SC on south-facing slopes (PC3) is controlled predominantly by the drivers of storms: the North Pacific and the Indian Ocean (NAO and DMI). This effect is not just related to cloud cover, but also depends on the amount of precipitation. Monsoon brings with it high humidity and temperature fluctuations, leading to snow depletion at lower altitudes (PC1). SC on high slopes (PC2) is relatively unaffected from depletion during monsoon, and hence, they display the fingerprints of variations in monsoon strength. The usual suspects for monsoon activity – the Indian Ocean, the north Pacific^98^, and the south Pacific – are confirmed to be the influencers of SC (DMI, PDO, Nino 1+2). High valleys (PC3) also reflect signatures of these climate drivers in the correlation analysis, but the best subset regression analysis shows the Arctic (AO) and North Atlantic (AO) to be the common drivers across all basins. This is likely due to the varying nature of a basin’s interactions with monsoon systems. Monsoon is, by and large, a time of snow depletion (left-over snow from the previous seasons), which is the reason why the dominant drivers of winter weather systems show up once again in monsoon. As monsoon winds retreat during fall, the drivers of monsoon remain as players. However, fall is also the season of declining temperatures; hence, it is a combination of remote forcings and local precipitation that guides SC expansion. The major region of change, the mid altitudes (PC1), show that the importance of the Southern Pacific (Nino 1+2) diminishes, while the Indian Ocean (DMI) still retains a role to play. The importance of the Arctic (AO) and North Pacific (PDO) increases, and this is reflected in the mountain slopes (PC3).

Archer & Fowler (2004)^83^ had claimed winter precipitation in their study area (west of ours) does not exhibit much spatial variation. Their data stations were all close to river channels, and our study suggests that they might have unknowingly been confined in the sphere of NAO influence. On the other hand, some broad patterns of spatial variability of precipitation were identified by Banerjee & Singh (2022)^99^ who used reanalysis data to establish several precipitation altering mechanisms at the 500 hPa atmospheric level in a study area that encompassed ours. The limitation of the study (Banerjee & Singh (2022)) is related to its comparison of SC between Indian administrative units, and this limitation is part of a tradition where spatial variability of SC area is only examined either at a regional scale^100,101^ (comparison across regions defined by climate zones), basin scale^102,103^ (comparisons across basins), or at best, across topographies^104,105^ (comparisons across elevation, slope and aspect classes). To the best of our knowledge, this is the first paper that lets Himalayan snow speak for itself, meaning, our integrated PCA and high-resolution satellite data groups areas by SC behavior and hence uncovers more fundamental geomorphological units influencing SC. In the global context, even though the PCA approach has been applied to analyze SC, these studies had only applied PCA on long but seasonally unsegregated time series^106–109^ – thus missing the intraseasonal SC variability. While an examination of the microclimates of these regions leading to similar behavior was beyond the scope of the present work, we established the spatially varied effect of various climate drivers on SC, and showed that climate drivers do not act uniformly across the entire river basins. Nevertheless, we acknowledge that our study was limited by a common problem of optical remote sensing, i.e., interruptions in observations by cloud cover (especially in monsoon and occasionally in winter). However, the observed seasonal nature of cloud cover led us to some of the explanations offered before. We intentionally omitted Zanskar basin (another important basin in the region) from our study as the basin was too large to calculate PCs with the computing power at our disposal. However, having considered river basins northwards and southwards of Zanskar, we are confident in our claim that the patterns we have presented here are general for the western Himalayas. An interested reader might ask why different Nino indices present themselves as drivers of (various) SC characteristics in each season (also recall that two different indices of the ENSO state drive two different characteristics of SC in the long term). We posit a partial explanation here: the ENSO might appear to be a single remote forcing agent; however, former studies^110^ showed that different parts of the South Pacific can play different roles in forming teleconnections with monsoon. Further, ENSO has been shown to operate through different teleconnection mechanisms in different seasons^111^. It should not come as a surprise that different climate variability modes drive different characteristics of SC in each season.

Sea surface temperatures change slowly, and therefore might be useful for forecasting SC fractions in Himalayan river basins – a traditionally difficult area for climate models, especially at a high resolution. SC fraction is an essential input in various hydrological models, which are of great importance in estimating water availability and subsequent water management. The climate indices can be used as predictors for an early warning system to forecast hydroclimatic conditions of the mountains at different lead times. Overall, our study has demonstrated that ocean–atmosphere couplings, along with local meteorological factors have a major impact on distinct components of SC extent in the Himalayas.

## Data and methods

Extended Landsat images (level 2, collection 2) from missions 4,5^112^,7^113^, 8, and 9^114^ from August-1989 to February-2025 were downloaded from the United States Geological Survey (USGS) earth explorer website^115,116^. Temporal resolution (i.e., the repeat coverage) of all the missions is 16 days, and the observations have a common spatial resolution of 30 m. The Landsat program was chosen as the data source as it offers the oldest continuous series of satellite images, and the temporal resolution is sufficient for our seasonal scale study. Daily gridded (0.25° × 0.25°) precipitation^117,118^ and gridded (1° × 1°) temperature^119^ (maximum^120^ and minimum^121^) were obtained from Climate Monitoring and Prediction Group (CMPG) web-portal of the India Meteorological Department. The ‘average temperature’ (referred to as temperature) was computed from the minimum and maximum temperatures. Global Satellite Radar Topography Mission (SRTM) Digital Elevation Model (DEM)^122^ downloaded from USGS Earth Explorer was used to calculate topographical parameters like elevation, slope, aspect, and then delineate six watersheds using QGIS^123^. Among the six basins, Spiti is the largest basin with an area of 12,393 km^2^, while Baspa is the smallest basin with an area of 998 km^2^. Details related to the basins, can be found in Table 1.

**Table 1:** Area, observation period, and number of available satellite observations after cloud cover elimination.

Basin	Landsat tiles used (path, row)	Area (Sqkm)	Time period	No. of monthly observations available (direct + proxy + averages)
Baspa	146, 038	997.9722	05/12/1989–01/11/2023	263 + 41 + 25 = 329
Beas	147, 038	5339.781	06/08/1989–16/11/2023	210 + 53 + 21 = 284
ChandraBhaga	148, 037; 147, 038; 147, 037	10,779.4413	01/08/1989–01/11/2023	148, 037: (262 + 48 + 35) 147, 038: (245 + 58 + 30) 147, 037: (264 + 46 + 27) Concatenated images = 306
Nubra	148, 035; 147, 036	4206.8637	01/12/1989–01/11/2023	148, 035: (244 + 38 + 54) 147, 036: (256 + 36 + 32) Concatenated images = 272
Ravi	147, 038	2627.973	06/08/1989–16/11/2023	240 + 46 + 34 = 320
Spiti	147, 037; 146, 037; 146, 038	12,393.297	01/12/1989–01/11/2023	147, 037: (262 + 40 + 31) 146, 037: (254 + 44 + 30) 146, 038: (258 + 47 + 19) Concatenated images = 283

As per the Köppen Classification System, the region largely comes under humid sub-tropical with dry winters (Cwb) in lower altitudes to subtropical highlands (Cfa) in higher altitudes^124^. The current study extracted relevant information from satellite images and meteorological datasets particularly for six basins based on basin boundaries. In addition, monthly values of climate variability modes, also informally referred to as oceanic-atmospheric indices (AO^125^ for Arctic Oscillations, NAO^126^ for North Atlantic Oscillations, PDO^127^ for Pacific Decadal Oscillations, SOI^128^, NINO 1+2^129^, NINO 3^130^, NINO 3.4^131^, and NINO 4^132^ for El Niño Southern Oscillations, and DMI^133^ – the Dipole Moment Index for Indian Ocean Dipole were downloaded from the NOAA repository. Subsequently, all variables were truncated for a common window of 1989–2025 (additional details discussed later).

All calculations were performed using R^134^ (unless otherwise specified) and its libraries – terra^135^, lubridate^136^, imputeTS^137^, openair^138^, circlize^139^, colorRamp2^140^ fields^141^, RcolorBrewer^142^, ComplexHeatmap^143^, and magick^144^. Normalized Difference Snow Index (NDSI) images were calculated from the Landsat scenes (for Landsat 7, scan line error related gaps were first interpolated spatially using QGIS). NDSI formulation based on spectral properties of snow at a particular pixel is given in Supplementary Eq. 1. Non-positive NDSI values were replaced with zero since the non-positive values hold no significance for SC calculation. Subsequently, images with cloud cover over the study area were discarded by visual inspection (following an initial threshold of 20% maximum cloud cover in the earth explorer website). This resulted in a reduction in the number of total observations, but enabled us to use a threshold of NDSI values greater than zero as the indicator of SC without confusing between snow and cloud. Note that when multiple images were available for a single month, the images were averaged to arrive at a monthly composite. Moreover, when no data was available for a month, but multiple observations were available in the preceding or subsequent month towards the end or the beginning respectively, those observations were counted as a proxy for the month with missing data. In cases when such proxies were also not available, and the gap was only a single month gap, an average of the preceding and subsequent months was used as a proxy observation, as SC trends are typically well behaved over monthly time scales. Other data gaps were left as they are except for wavelet analysis, where a Stineman interpolation^145^ was performed on the final SC parameters as wavelet analysis requires uniform time steps. Typically, if a basin is large enough to not be covered by a single flight path, we concatenated multiple Landsat images within a month to develop a composite image for a basin (after filling data gaps for each Landsat tile following the approach previously described). Even after image-processing, if a data gap exists for a particular month, subsequent meteorological and climate variability modes for the months were discarded from further analysis. Details of the length of the observations are presented in Table 1.

Three characteristics of SC were calculated based on NDSI images for each basin: FSC, TSC, and the PCs of NDSI. FSC, a common attribute in cryosphere studies, was calculated as the fraction of the basin area under SC^146^ (Supplementary Eq. 2). The area under SC was calculated by counting the number of pixels in a basin with NDSI values greater than zero. Note that FSC only captures the instantaneous SC; hence, it does not represent the SC that sustains for a longer period like a season. Note that FSC only captures the instantaneous SC and hence, it cannot distinguish between snow that sustains through an entire season and snow that melts within a season. This variable part (i.e., the sub-seasonal variability of SC), however, is of immense hydrological and ecological significance, especially on a seasonal to sub-seasonal scale, as it contributes a substantial part of the snow-water equivalent always available in a snow-dominated watershed. Hence, we propose a new metric: TSC, calculated as the fractional area of a basin that is under SC at a given time, but does not retain SC for the entire season (Eq. 1).

1 $$\begin{aligned} & Fractional \,area\, covered\, in\, temporary\, snow\, (TSC)\\ & \quad=\frac{Snow\, covered\, area\,-Area\, under\, snow\, cover\, for\, entire\, season\,}{Area\, of\, the\, Basin\,}\end{aligned}$$

We note that FSC and TSC are aggregated measures that give a single value for an entire watershed. Despite their computational simplicity, FSC and TSC cannot comprehensively yield the spatio–temporal dynamics of SC in the complex geography the western Himalayas offer. To retain the spatio–temporal characteristics of SC variability, we conducted a principal component analysis^147^. In principal component analysis, pixels of a watershed are treated as variables which take on values of the observed NDSI timeseries. The principal component technique creates different linear combinations of the pixels (variables), and each of these linear combinations (Principal Components – PCs) capture an independent mode of variability (the first PC captures the major part of the variability). The pixels which exhibit more variance (towards a variability mode) are given higher weights in the corresponding linear combination (PC) – this enables us to identify spatial regions which behave similarly towards contributing towards a mode of temporal variability. Note that two separate analyses are carried out – the first with the entire time series, and the second with seasonal segregation based on four seasons. An example data matrix for PC analysis is given in Supplementary Eq. 3.

To examine the long-term controls on SC variability, wavelet coherence analysis^148,149^ was conducted^150^ based on the five SC attributes and various climate variability modes. In wavelet coherence analysis of two time series (in our case, combinations of a SC attribute and a climate variability mode), each of them is treated as a combination of periodic signals, or waves of various wavelengths. It is then checked, from shorter to longer time scales (wavelengths) if there is a coherence (essentially correlation for waves) between the two waves. Two waves can move in phase, implying that the two phenomena occur together, or with some phase difference, indicating one phenomenon precedes the other. This analysis shows if two phenomena had coherence for some period of time at some time–frequency (i.e., wavelength). Additional details related to the wavelet coherence analysis are presented in Supplementary Eq. 4.

Further, we note that the study area undergoes intense seasonal changes; hence, the entire time series of the dependent and independent variables were split into seasonal subsets. Four seasons were considered as Winter (December-January–February), Summer (March–April-May), Monsoon (June-July–August-September), and Fall (October–November). This choice of seasonal segregation is to keep consistency with the standard practice of hydro-cryosphere research in the Indian subcontinent. Furthermore, to examine the seasonal controls of SC behavior, multiple correlation analysis (Pearson, Spearman, and Kendall) was performed on each SC parameter and climate variability modes (Supplementary Eqs. 5.1–5.3). Correlations were calculated not only for concurrent observations of the variable pairs, but also with lagged versions of the climate variability indices (e.g. a one-month lag implies correlation between an observed SC parameter for a month and an observed climate variability index from the previous month). We note that lagged correlations allowed us to explore processes that operate over longer time scales. While Pearson correlation can yield the linear association between two variables, Spearman and Kendall are rank correlations that identify non-linear but monotonic associations. Further, to assess model sensitivity we calculated upper and lower bounds to the correlation coefficients using the Fisher Z transform^151,152^. Additionally, we did a composite analysis^153,154^ to validate the correlations observed – for each season and climate variability mode, we picked only the SC observations which were paired with the relevant climate variability index values outside of their 1 standard deviation range, and repeated the correlation analysis. True associations between SC and climate variability indices should be amplified during heightened activity periods of the corresponding climate variability modes (defined here has index value outside its 1 standard deviation range). In a final analysis, to understand how all hydro-climatic controls combinedly influence SC, and whether any or some of them have a more pronounced effect on the SC, we perform a best subset regression^155,156^. Towards this, a complete linear model with all independent parameters is considered, and then we check whether a smaller subset of independent variables better explains the variance of the dependent variable (more details in Supplementary Eqs. 6, 7.1, and 7.2). Note that prior to performing best subset regression, all the explanatory and target variables were normalized to a 0–1 range (Supplementary Eq. 8). Normalization ensures that any of the explanatory variables do not have an outsized effect on the regression model solely due to its numerical magnitude. Further, since normalization brings all variables to the same scale, the final regression coefficients can be interpreted as the relative effect any particular explanatory variable has on snow cover, in the presence of all the other explanatory variables in the model^157^. Due to the prohibitively large number of potentially independent variables, the best subset regression algorithm was run first with only the local meteorological variables and their 12 lags, then in the selected model zero and odd lags were added, and in the resulting model from this stage, finally, the even lags were added. The final/intermediate model was selected, based on Mallow’s C_p_, as the one which explains the most variance with the least variables^158^. Once again, to account for model sensitivity, we calculated the uncertainty of the regression coefficients^159^ (at 95% confidence interval).

In this analysis, two local parameters – monthly precipitation and temperature are considered in the mix of independent variables along with the climate variability modes, to develop a comprehensive list of potential drivers. We expect immediate controls like precipitation and temperature to be dominant controls on SC. If external parameters receive higher importance (coefficients of the parameters in the final linear model), there needs to be a good explanation.

## Supplementary Information

Supplementary Information.

## Data Availability

Landsat data can be downloaded from the USGS Earth explorer tool website (https://earthexplorer.usgs.gov/). Rainfall data can be downloaded from the IMD website (https://imdpune.gov.in/cmpg/Griddata/Rainfall_25_NetCDF.html), as can be minimum temperature (https://www.imdpune.gov.in/cmpg/Griddata/Min_1_Bin.html) and maximum temperature (https://www.imdpune.gov.in/cmpg/Griddata/Max_1_Bin.html). Indices used in the study can be obtained from the NOAA climate indices web page (https://psl.noaa.gov/data/climateindices/list/). Time series of FSC, TSC, and PCs for each basin, and the basin shapefiles used in the study have been made available at Zenodo (10.5281/zenodo.16406237). Contact the corresponding author for further data requests.
