# Publisher Correction: How seas whisper to snow: teleconnections drive spatio–temporal variability of snow cover in Western Himalayas

**DOI:** 10.1038/s41598-025-32540-7

**Published:** 2025-12-18

**Authors:** Shairik Sengupta, Rajarshi Das Bhowmik

**Affiliations:** Interdisciplinary Center for Water Research, Indian Institute of Science, Bengaluru, 560012 India

Correction to: *Scientific Reports* 10.1038/s41598-025-18606-6, published online 06 October 2025.

The original HTML version of this Article displayed an error in the names of Shairik Sengupta and Rajarshi Das Bhowmik, which were incorrectly given as Sengupta Shairik and Bhowmik Rajarshi Das.

The PDF version was correct at the time of publication.

The original Article has been corrected.

