# Supplementary material for: How seas whisper to snow: teleconnections drive spatio–temporal variability of snow cover in Western Himalayas
Source: Sci Rep. 2025 Oct 6;15:34787. doi: 10.1038/s41598-025-18606-6 (PMC12500974; doi:10.1038/s41598-025-18606-6)
Supplement: Supplementary file 1 — Supplementary Information. [file 41598_2025_18606_MOESM1_ESM.pdf]

Supplementary Information for

How Seas Whisper to Snow: Teleconnections Drive Spatial-temporal Variability of Snow Cover in Western Himalayas

Shairik Sengupta[1], Rajarshi Das Bhowmik[1]
1 Interdisciplinary Center for Water Research, Indian Institute of Science, Bengaluru

Contents

Figures: Supplementary Figures 1 to 18
Equations and explanations: Equations 1 to 7
Table: Supplementary Tables 1 to 2
Additional text: Supplementary Text 1

Figures

Supplementary Figure 1: Climatology of first three Principal Components of each basin

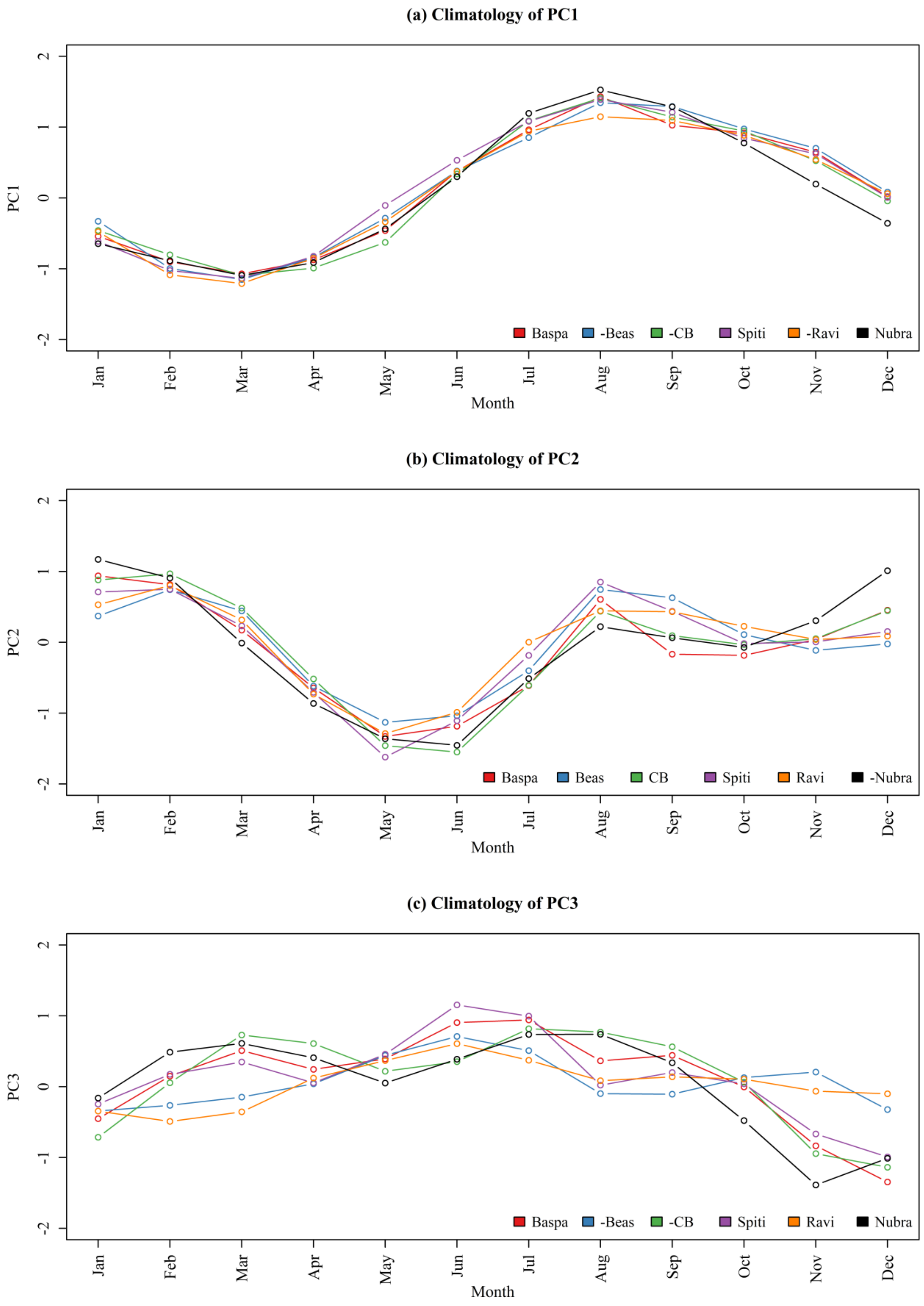

Supplementary Figure 1: Climatology of the first three principal components of each basin. Subplot (a) shows PC1, Subplots (b) and (c) depict PC2 and PC3 respectively. Signs of Principal Components are arbitrary, cases where the negative of a pc have been plotted to highlight the common climatological pattern across basins are marked in the legend with a

negative sign before the basin name. Each Principal Component for each basin has been normalized with standard scores before calculating climatology. In all subplots, Baspa is shown in red, Beas in blue, Chandrabhaga in green, Spiti in purple, Ravi in orange, and Nubra in black.

Supplementary Figure 2: Topography of basins

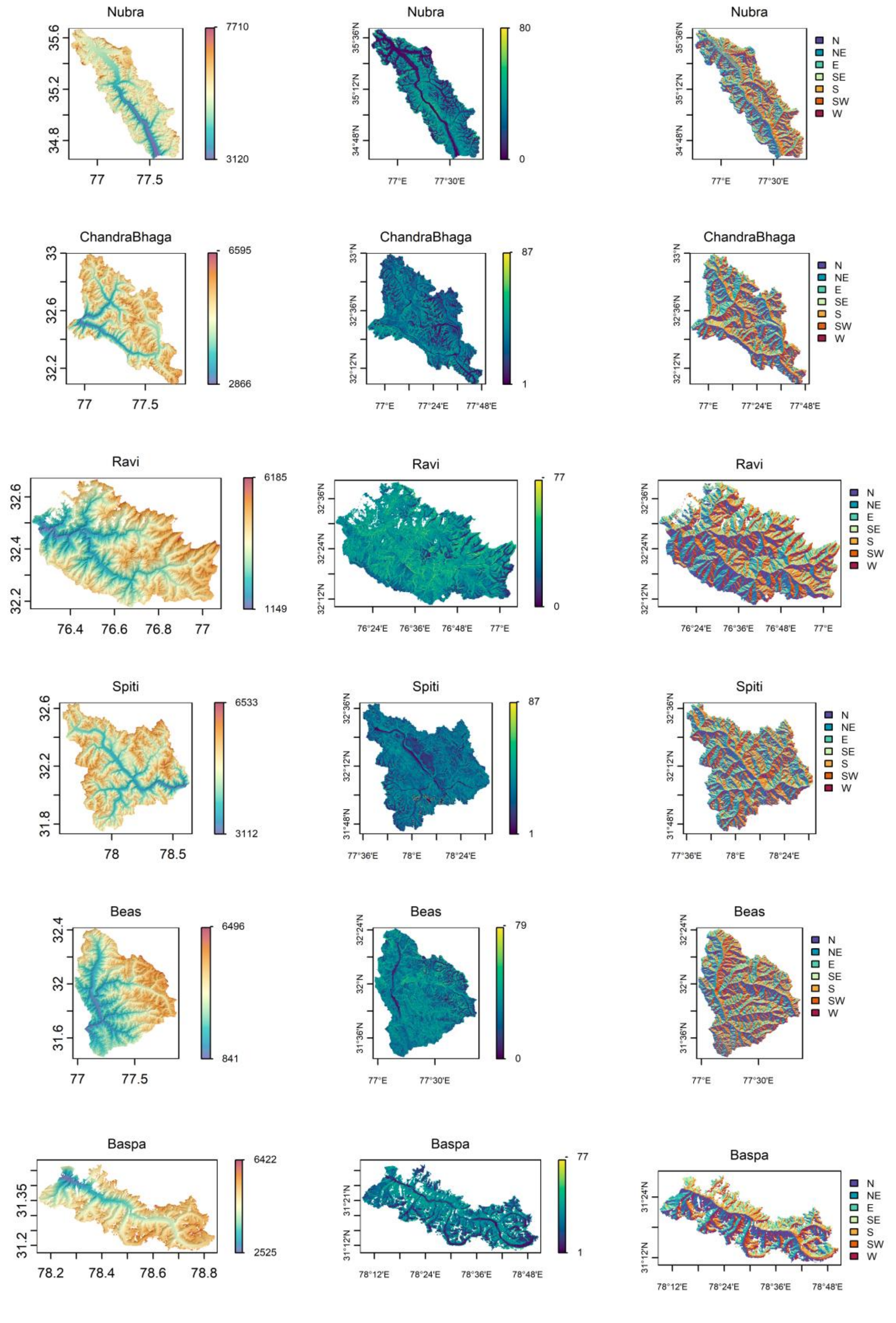

(a) Elevation (b) Slope (c) Aspect

Supplementary Figure 2: Topography of the river basins. Column (a) describes the elevation of the basins in meters. The legend depicts lower elevations in shades of blue and higher elevations in shades of red. Column (b) shows the slopes in degrees. Here a violet to yellow legend has been used. Column (c) shows the aspect in compass directions.

Supplementary Figure 3: Climatology of FSC, TSC, and observed precipitation and temperature for each basin

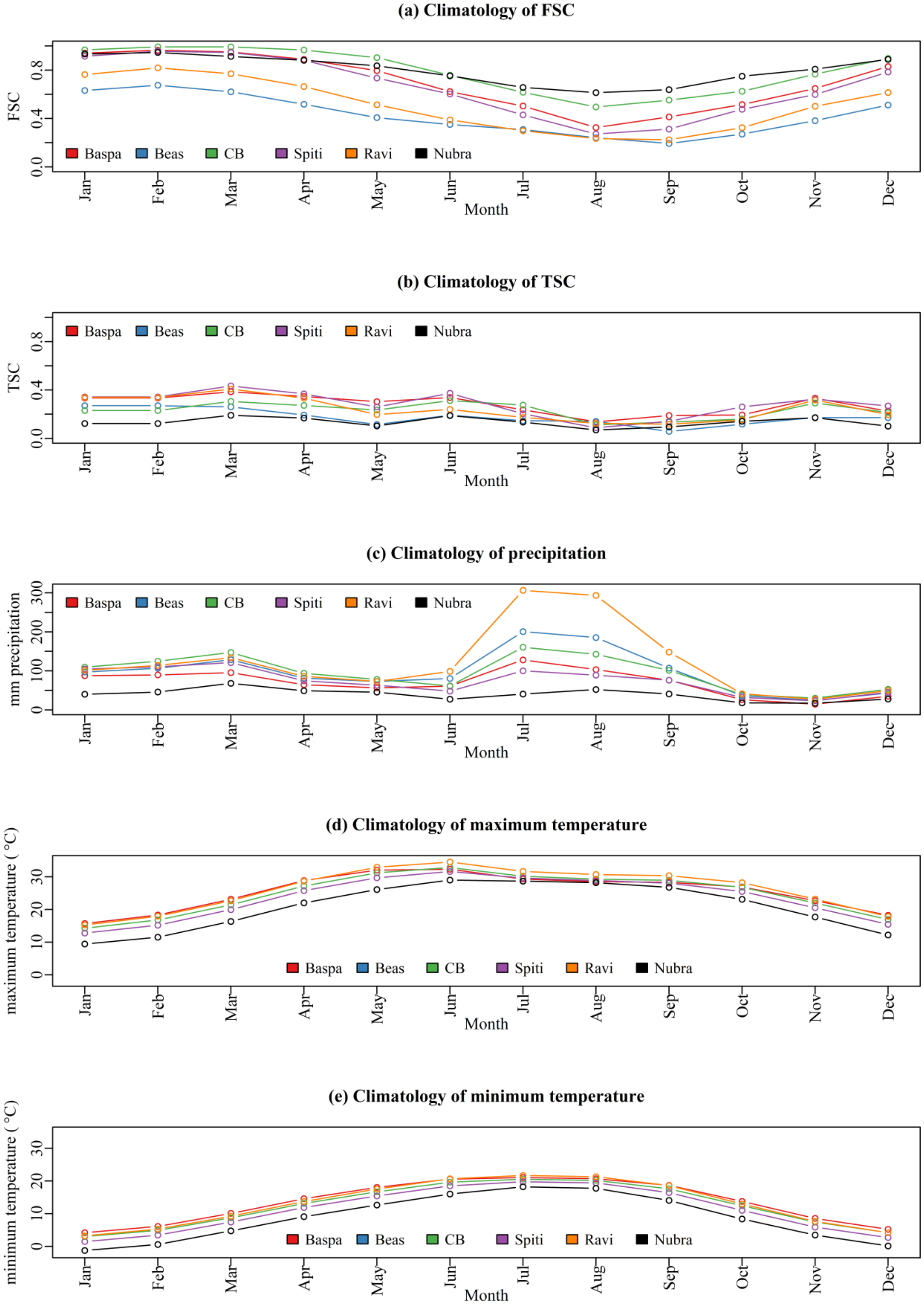

Supplementary Figure 3: Climatologies of FSC (subplot a), TSC (subplot b), precipitation (subplot c), maximum temperature (subplot d), and minimum temperature (subplot e). In all

subplots, Baspa is shown in red, Beas in blue, Chandrabhaga in green, Spiti in purple, Ravi in orange, and Nubra in black.

Supplementary Figure 4: Loadings of first three PCs, Beas basin

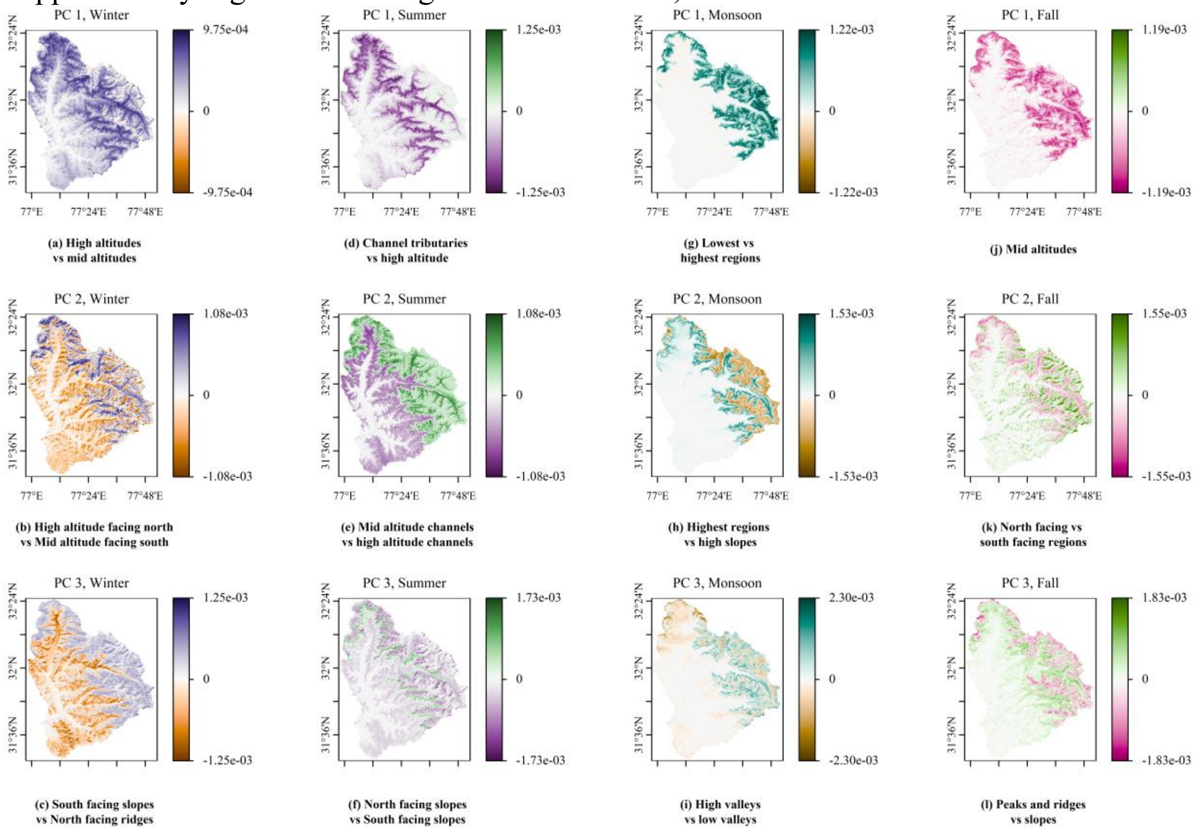

Supplementary Figure 4: Loadings of the first three principal components of snow cover variations in each season for Beas basin. Subplots a-c shows loadings of PC1-PC3 of winter; high positive values appear dark blue while high negative values appear orange. The loadings of the first three PCs of summer are given in subplots d-f. Dark green represents high positive values, dark purple stands for high negative values. PC1-PC3 loadings for monsoon are displayed in subplots g-i. Once again dark green depicts high positive values, while high negative values take dark on orange shades. In subplots j-l the loadings of PC1-PC3 of fall are shown. Dark green stands for high positive values, dark pink implies high negative values.

Supplementary Figure 5: Loadings of first three PCs, ChandraBhaga basin

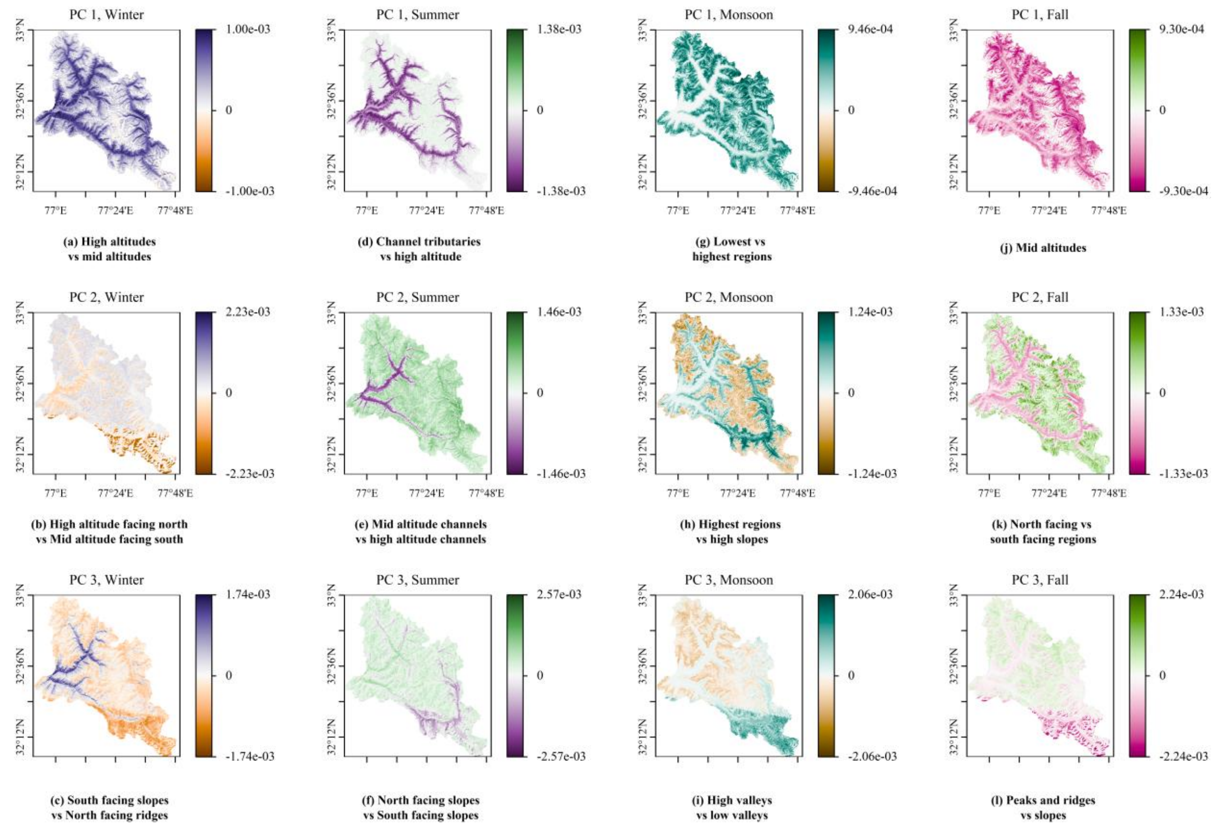

Supplementary Figure 5: Loadings of the first three principal components of snow cover variations in each season for ChandraBhaga basin. Subplots a-c shows loadings of PC1-PC3 of winter; high positive values appear dark blue while high negative values appear orange. The loadings of the first three PCs of summer are given in subplots d-f. Dark green represents high positive values, dark purple stands for high negative values. PC1-PC3 loadings for monsoon are displayed in subplots g-i. Once again dark green depicts high positive values, while high negative values take dark on orange shades. In subplots j-l the loadings of PC1-PC3 of fall are shown. Dark green stands for high positive values, dark pink implies high negative values.

Supplementary Figure 6: Loadings of first three PCs, Nubra basin

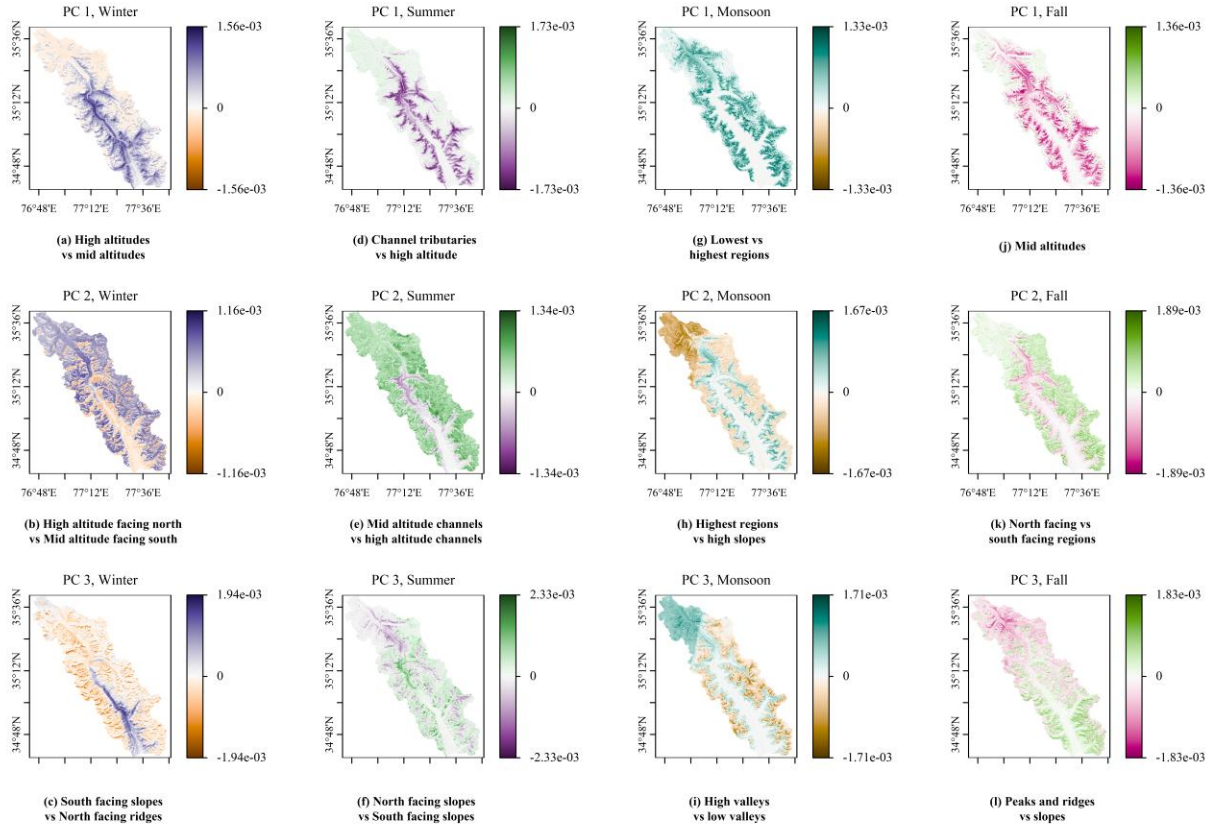

Supplementary Figure 6: Loadings of the first three principal components of snow cover variations in each season for Nubra basin. Subplots a-c shows loadings of PC1-PC3 of winter; high positive values appear dark blue while high negative values appear orange. The loadings of the first three PCs of summer are given in subplots d-f. Dark green represents high positive values, dark purple stands for high negative values. PC1-PC3 loadings for monsoon are displayed in subplots g-i. Once again dark green depicts high positive values, while high negative values take dark on orange shades. In subplots j-l the loadings of PC1-PC3 of fall are shown. Dark green stands for high positive values, dark pink implies high negative values.

Supplementary Figure 7: Loadings of first three PCs, Ravi basin

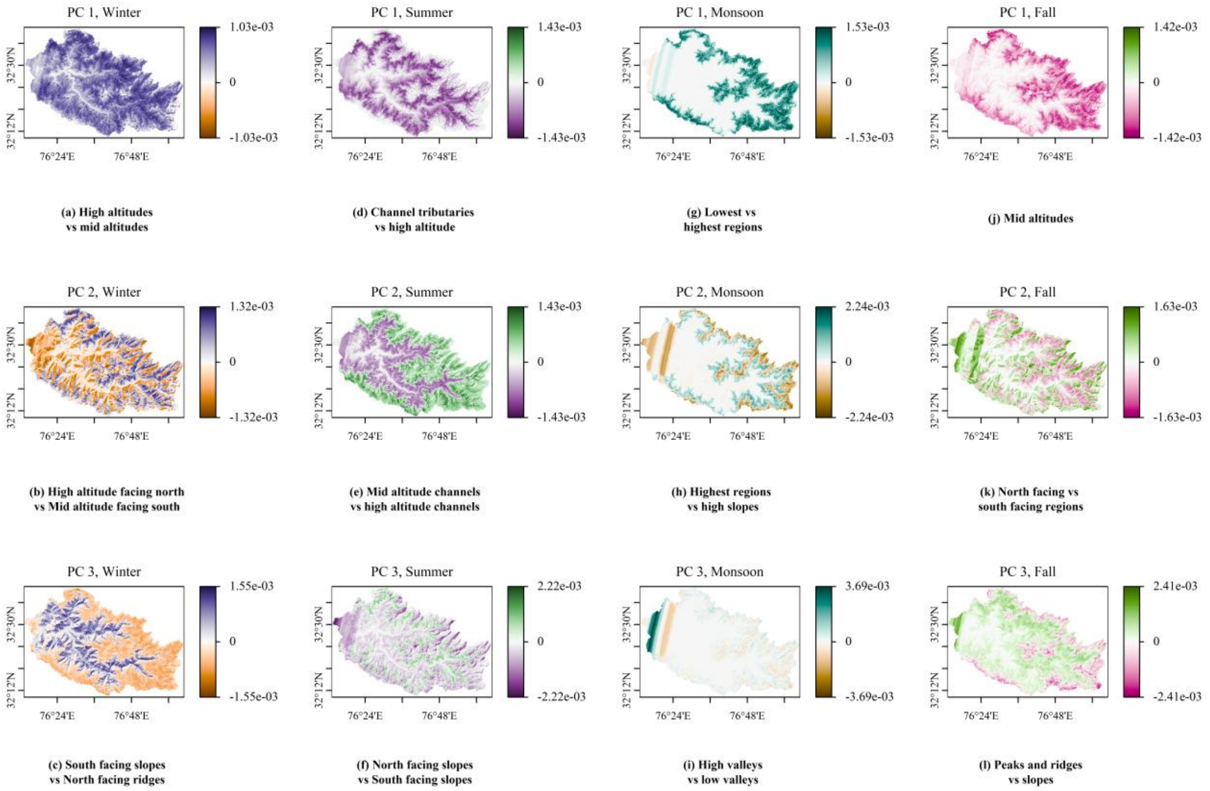

Supplementary Figure 7: Loadings of the first three principal components of snow cover variations in each season for Ravi basin. Subplots a-c shows loadings of PC1-PC3 of winter; high positive values appear dark blue while high negative values appear orange. The loadings of the first three PCs of summer are given in subplots d-f. Dark green represents high positive values, dark purple stands for high negative values. PC1-PC3 loadings for monsoon are displayed in subplots g-i. Once again dark green depicts high positive values, while high negative values take dark on orange shades. In subplots j-l the loadings of PC1-PC3 of fall are shown. Dark green stands for high positive values, dark pink implies high negative values.

Supplementary Figure 8: Loadings of first three PCs, Spiti basin

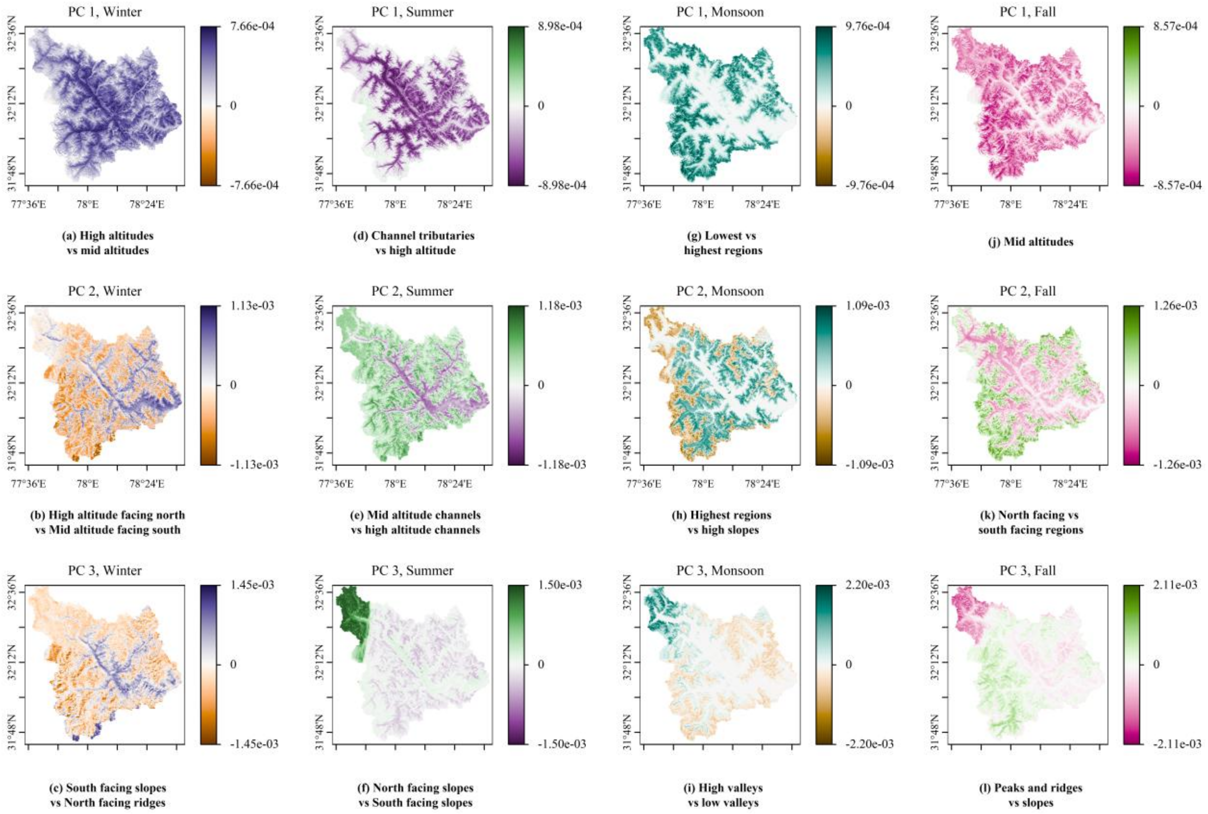

Supplementary Figure 8: Loadings of the first three principal components of snow cover variations in each season for Spiti basin. Subplots a-c shows loadings of PC1-PC3 of winter; high positive values appear dark blue while high negative values appear orange. The loadings of the first three PCs of summer are given in subplots d-f. Dark green represents high positive values, dark purple stands for high negative values. PC1-PC3 loadings for monsoon are displayed in subplots g-i. Once again dark green depicts high positive values, while high negative values take dark on orange shades. In subplots j-l the loadings of PC1-PC3 of fall are shown. Dark green stands for high positive values, dark pink implies high negative values.

Supplementary Figure 9: Lower bounds for estimated correlation coefficients between oceanic-atmospheric indices and snow cover parameters for 95% confidence interval.

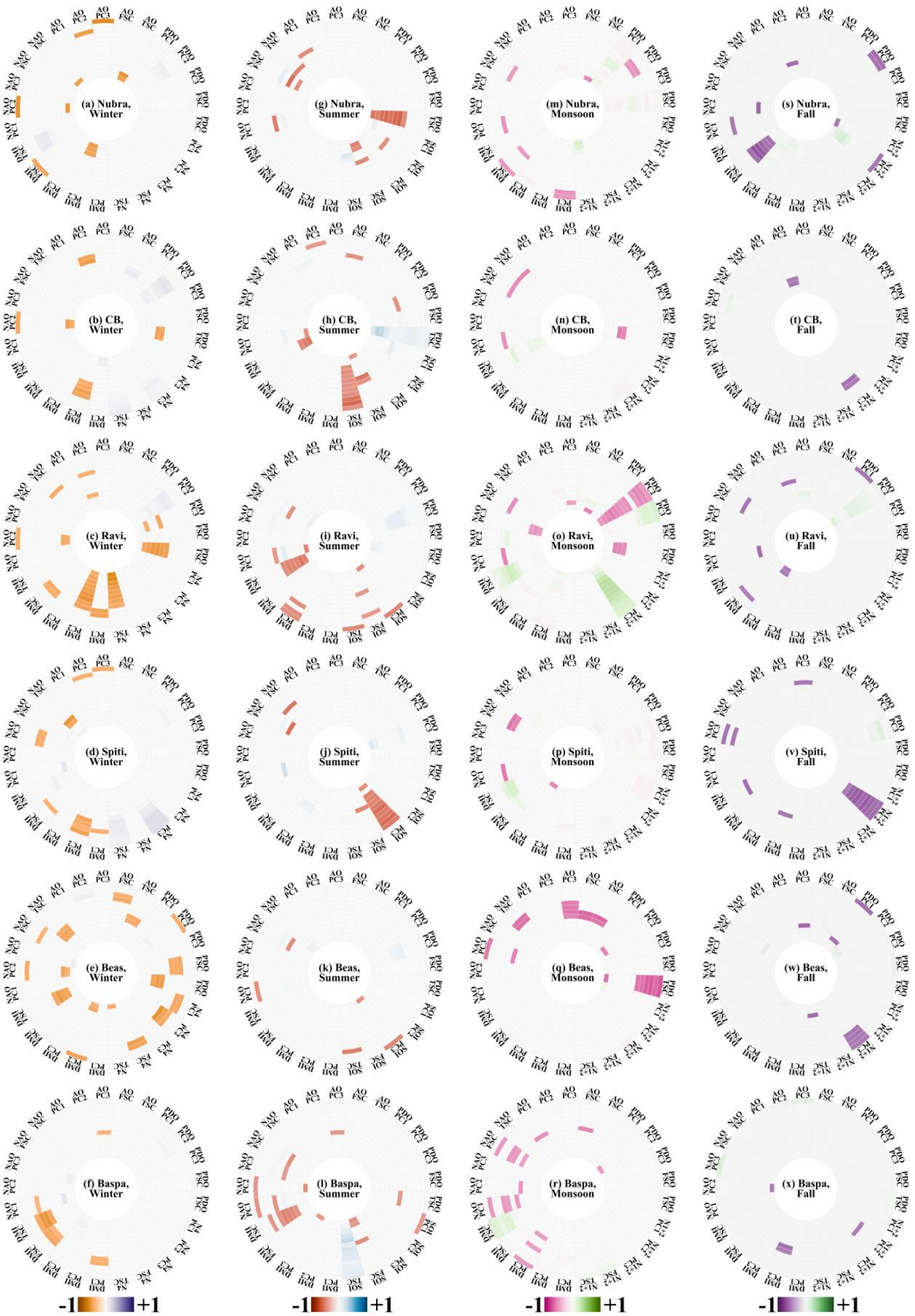

Supplementary Figure 9: Lower bounds to correlation (Pearson's r) between snow cover parameters (first three principal components of snow cover, fractional snow cover, and fractional temporary snow cover) and potential drivers (oceanic-atmospheric indices). In all

the plots, the circle closest to center represents the lower bound to correlation at lag 0. Concentric circles outwards stand for increasing lags of oceanic-atmospheric indices, with the final perimeter circle standing for lag 12. Each circle is divided into 25 sectors – combinations of five oceanic-atmospheric indices and the five snow cover parameters that were tested for correlation. These combinations are denoted outside the perimeter. For winter (subplots a-f), the legend runs from orange to purple, denoting -1 to +1. The same range of values are represented with a red to blue legend for summer (subplots g-l). A red to green legend is used to depict the lower bounds obtained for monsoon (subplots m-r). For fall finally (subplots s-x), lower bound values are shown with a purple to green legend.

Supplementary Figure 10: Upper bounds for estimated correlation coefficients between oceanic-atmospheric indices and snow cover parameters for 95% confidence interval.

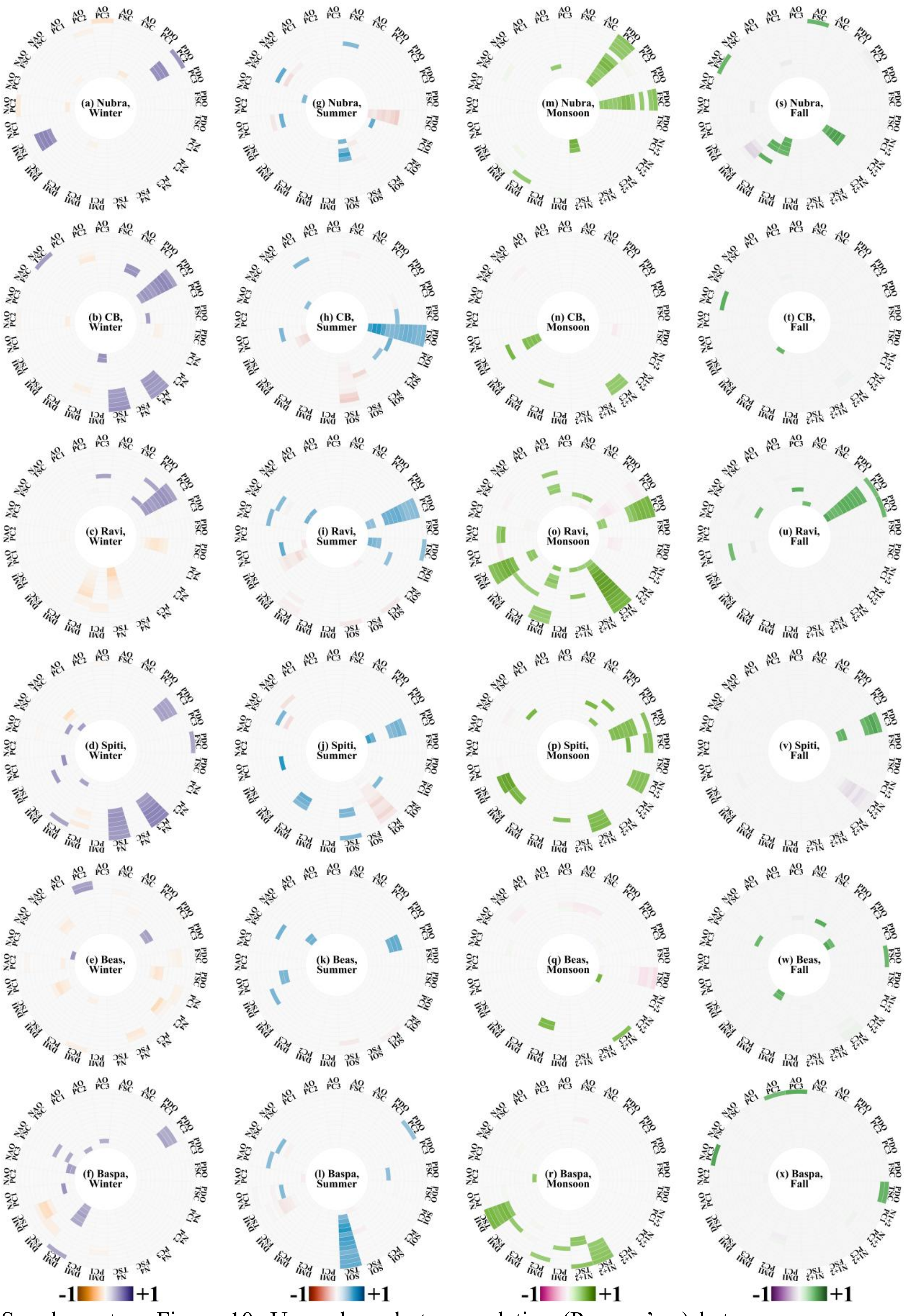

Supplementary Figure 10: Upper bounds to correlation (Pearson's r) between snow cover parameters (first three principal components of snow cover, fractional snow cover, and fractional temporary snow cover) and potential drivers (oceanic-atmospheric indices). In all

the plots, the circle closest to center represents the upper bound to correlation at lag 0. Concentric circles outwards stand for increasing lags of oceanic-atmospheric indices, with the final perimeter circle standing for lag 12. Each circle is divided into 25 sectors – combinations of five oceanic-atmospheric indices and the five snow cover parameters that were tested for correlation. These combinations are denoted outside the perimeter. For winter (subplots a-f), the legend runs from orange to purple, denoting -1 to +1. The same range of values are represented with a red to blue legend for summer (subplots g-l). A red to green legend is used to depict the upper bounds obtained for monsoon (subplots m-r). For fall finally (subplots s-x), upper bound values are shown with a purple to green legend.

Supplementary Figure 11: Non-linear correlation (Kendall's τ) between snow cover parameters and climate variability modes

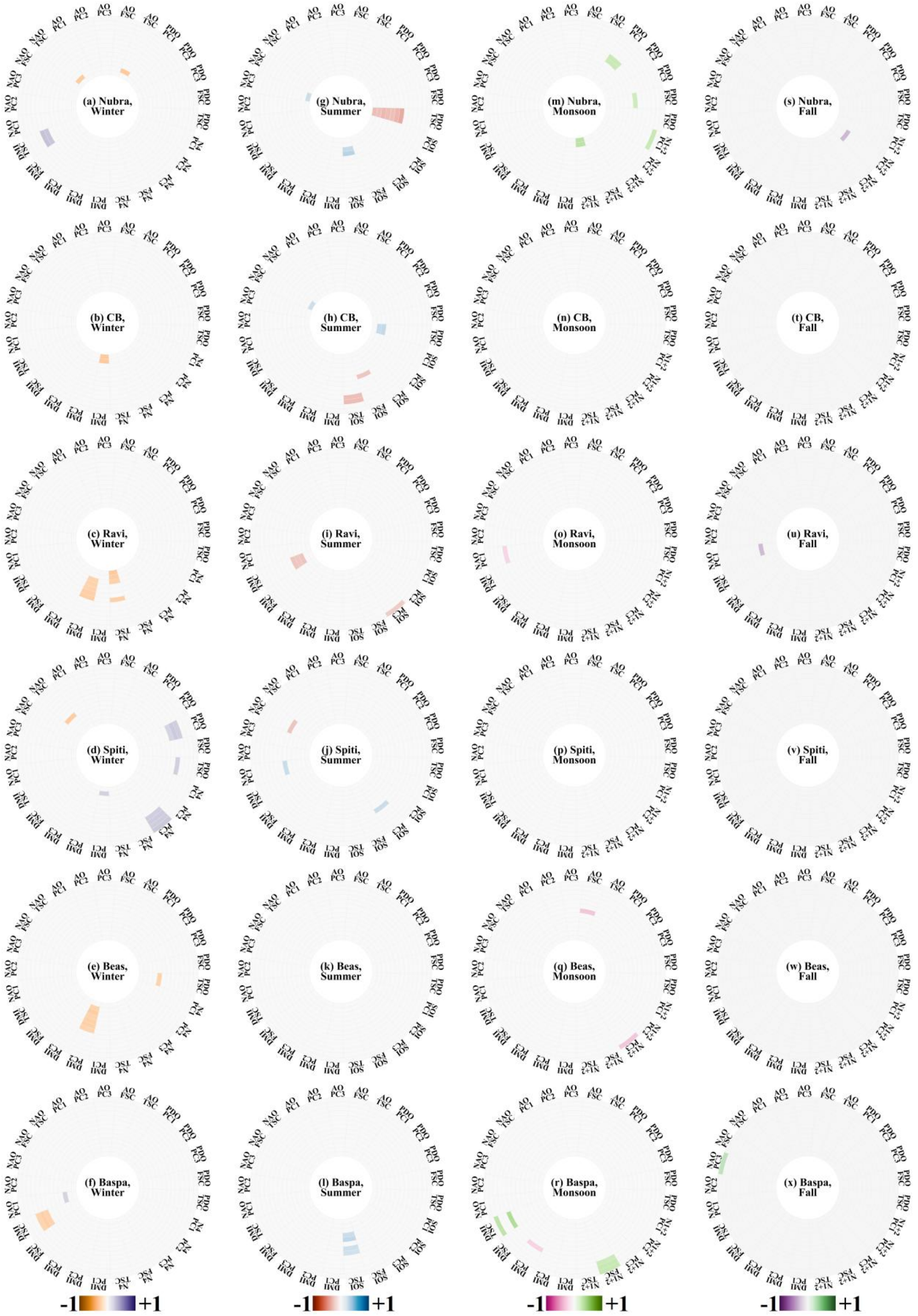

Supplementary Figure 11: Correlation (Kendall's τ) between snow cover parameters (first three principal components of snow cover, fractional snow cover, and fractional temporary snow cover) and potential drivers (oceanic-atmospheric indices). In all the plots, the circle closest to

centre represents the correlation at lag 0. Concentric circles outwards stand for increasing lags of oceanic-atmospheric indices, with the final perimeter circle standing for lag 12. Each circle is divided into 25 sectors – combinations of five oceanic-atmospheric indices and the five snow cover parameters that were tested for correlation. These combinations are denoted outside the perimeter. For winter (subplots a-f), the legend runs from orange to purple, denoting -1 to +1. The same range of values are represented with a red to blue legend for summer (subplots g-l). A red to green legend is used to depict the r values obtained for monsoon (subplots m-r). For fall finally (subplots s-x), r values are shown with a purple to green legend.

Supplementary Figure 12: Correlation coefficients between oceanic-atmospheric indices and snow cover parameters, during periods of AO index outside of its 1standard deviation range

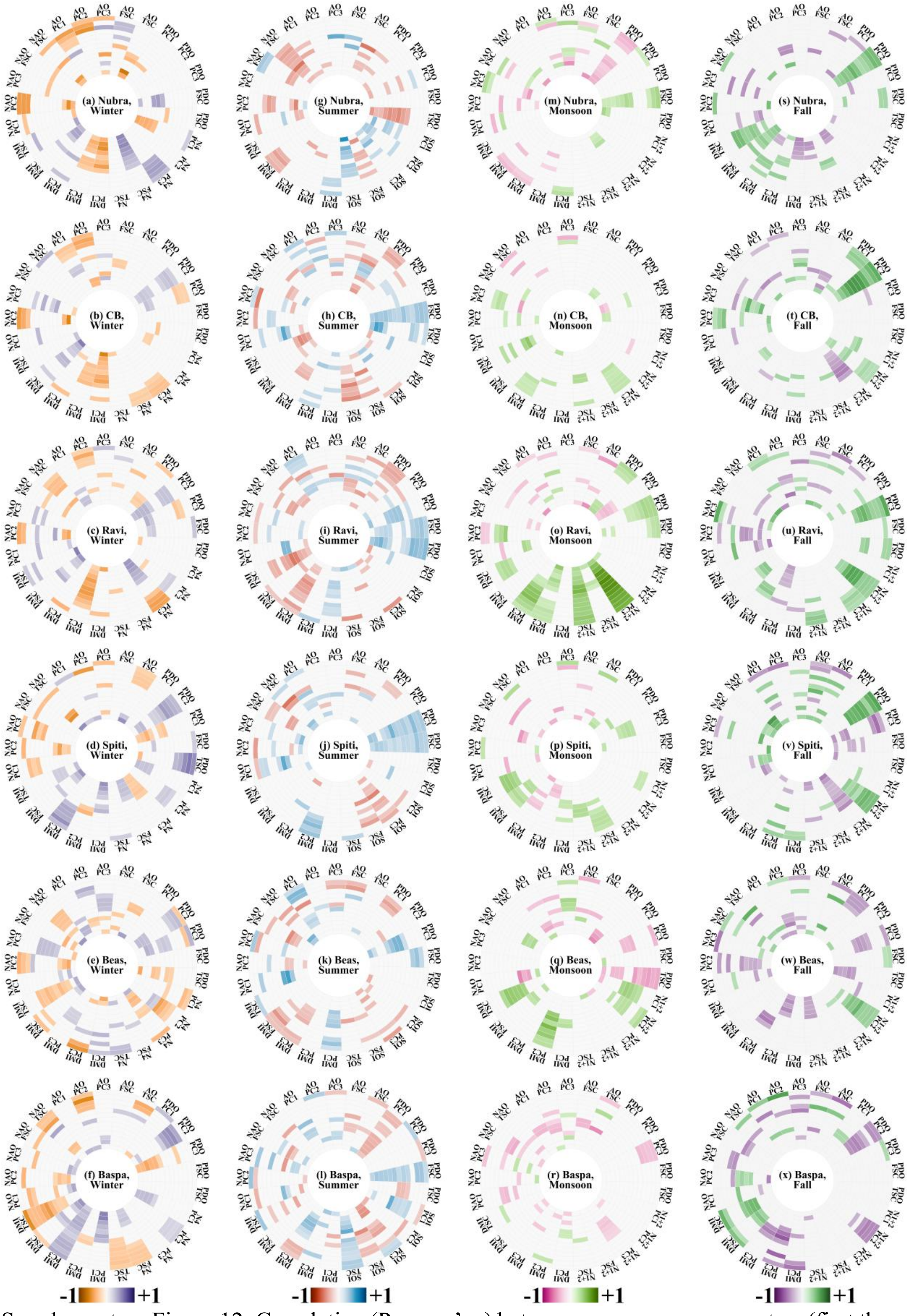

Supplementary Figure 12: Correlation (Pearson's r) between snow cover parameters (first three principal components of snow cover, fractional snow cover, and fractional temporary snow cover) and potential drivers (oceanic-atmospheric indices) - during periods of AO index outside

of its 1standard deviation range. In all the plots, the circle closest to center represents the correlation at lag 0. Concentric circles outwards stand for increasing lags of oceanic-atmospheric indices, with the final perimeter circle standing for lag 12. Each circle is divided into 25 sectors – combinations of five oceanic-atmospheric indices and the five snow cover parameters that were tested for correlation. These combinations are denoted outside the perimeter. For winter (subplots a-f), the legend runs from orange to purple, denoting -1 to +1. The same range of values are represented with a red to blue legend for summer (subplots g-l). A red to green legend is used to depict the r values obtained for monsoon (subplots m-r). For fall finally (subplots s-x), r values are shown with a purple to green legend.

Supplementary Figure 13: Correlation coefficients between oceanic-atmospheric indices and snow cover parameters, during periods of NAO index outside of its 1standard deviation range

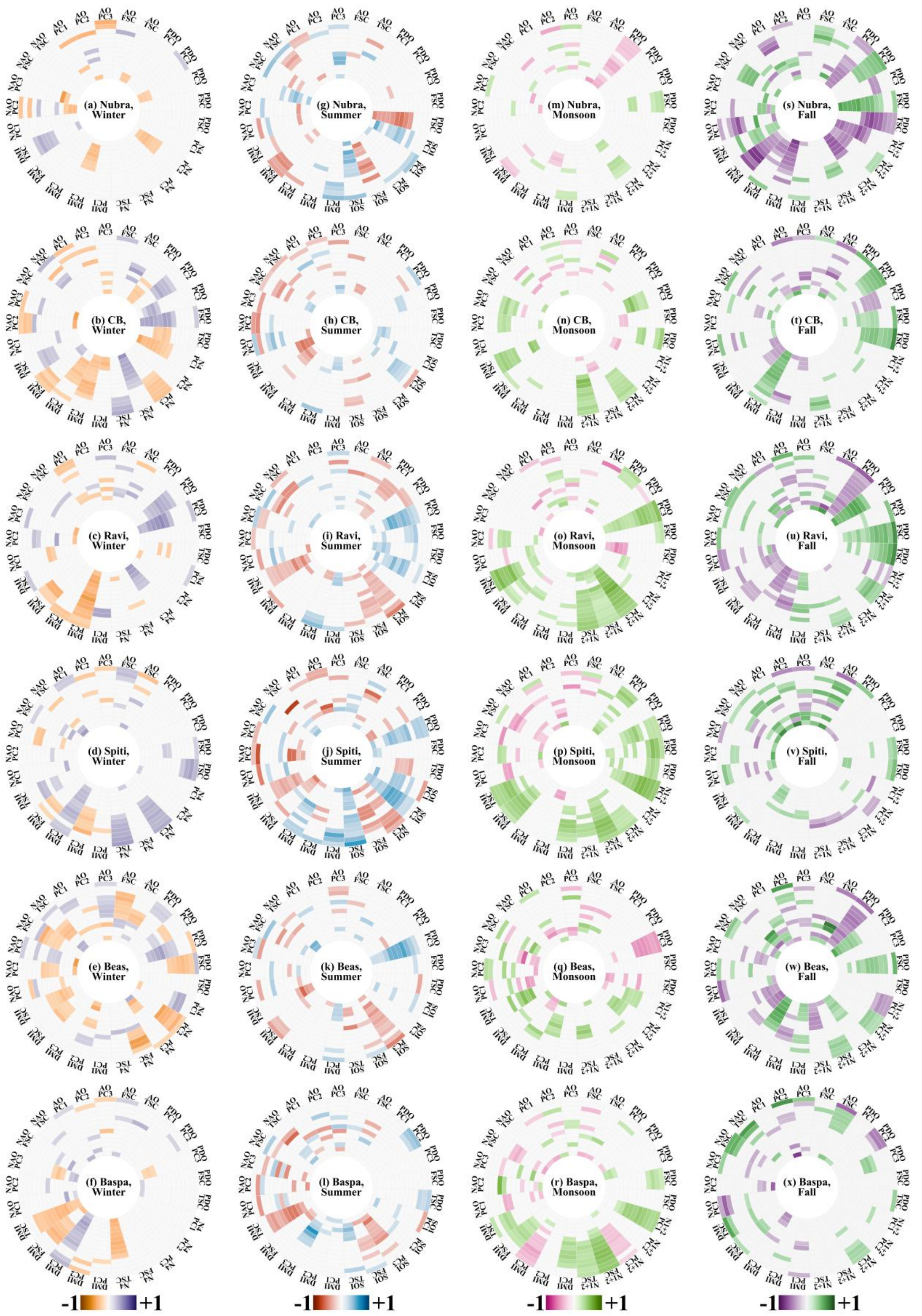

Supplementary Figure 13: Correlation (Pearson's r) between snow cover parameters (first three principal components of snow cover, fractional snow cover, and fractional temporary snow cover) and potential drivers (oceanic-atmospheric indices) - during periods of NAO index

outside of its 1standard deviation range. In all the plots, the circle closest to center represents the correlation at lag 0. Concentric circles outwards stand for increasing lags of oceanic-atmospheric indices, with the final perimeter circle standing for lag 12. Each circle is divided into 25 sectors – combinations of five oceanic-atmospheric indices and the five snow cover parameters that were tested for correlation. These combinations are denoted outside the perimeter. For winter (subplots a-f), the legend runs from orange to purple, denoting -1 to +1. The same range of values are represented with a red to blue legend for summer (subplots g-l). A red to green legend is used to depict the r values obtained for monsoon (subplots m-r). For fall finally (subplots s-x), r values are shown with a purple to green legend.

Supplementary Figure 14: Correlation coefficients between oceanic-atmospheric indices and snow cover parameters, during periods of PDO index outside of its 1standard deviation range

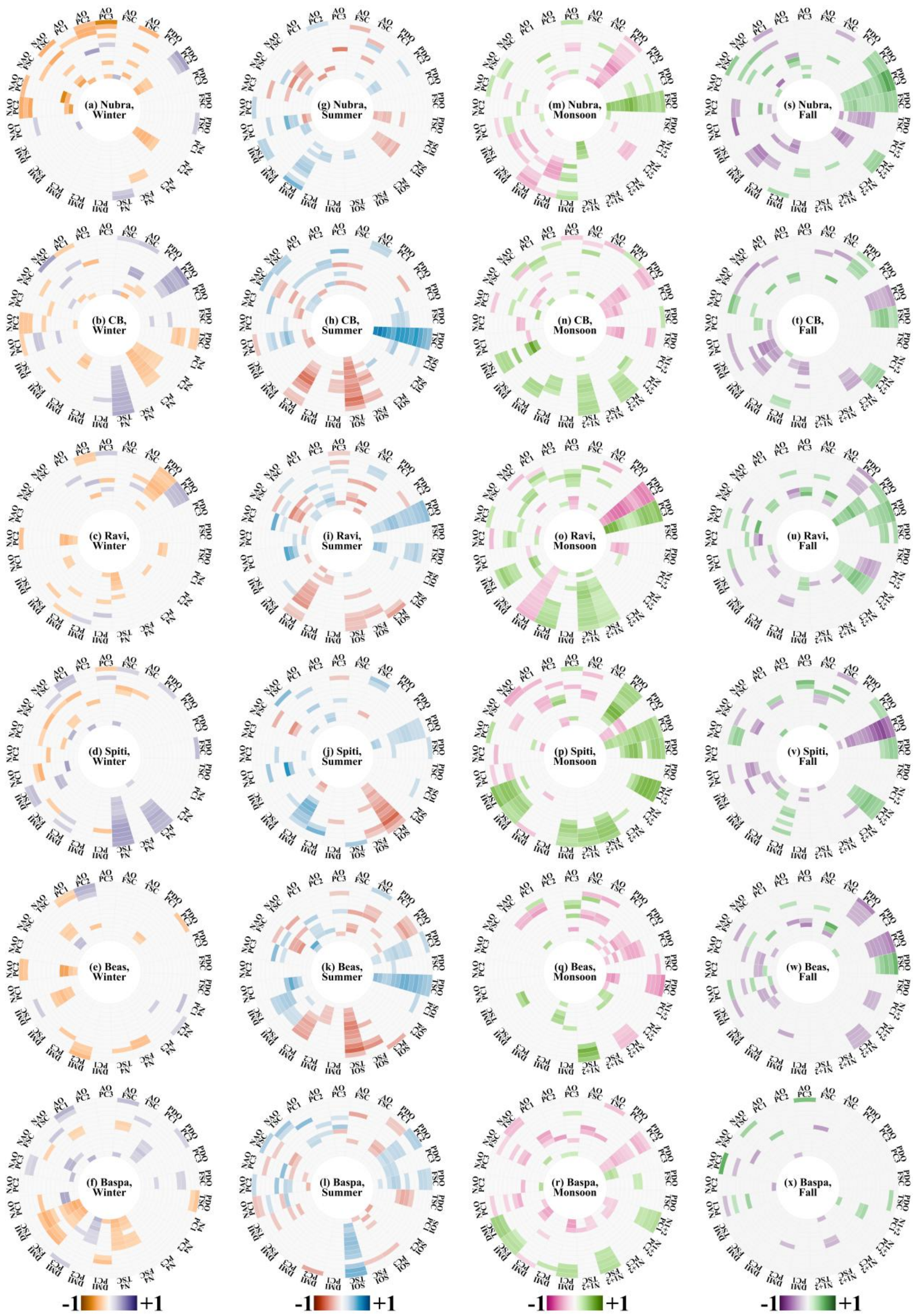

Supplementary Figure 14: Correlation (Pearson's r) between snow cover parameters (first three principal components of snow cover, fractional snow cover, and fractional temporary snow cover) and potential drivers (oceanic-atmospheric indices) - during periods of PDO index

outside of its 1standard deviation range. In all the plots, the circle closest to center represents the correlation at lag 0. Concentric circles outwards stand for increasing lags of oceanic-atmospheric indices, with the final perimeter circle standing for lag 12. Each circle is divided into 25 sectors – combinations of five oceanic-atmospheric indices and the five snow cover parameters that were tested for correlation. These combinations are denoted outside the perimeter. For winter (subplots a-f), the legend runs from orange to purple, denoting -1 to +1. The same range of values are represented with a red to blue legend for summer (subplots g-l). A red to green legend is used to depict the r values obtained for monsoon (subplots m-r). For fall finally (subplots s-x), r values are shown with a purple to green legend.

Supplementary Figure 15: Correlation coefficients between oceanic-atmospheric indices and snow cover parameters, during periods of DMI outside of its 1standard deviation range

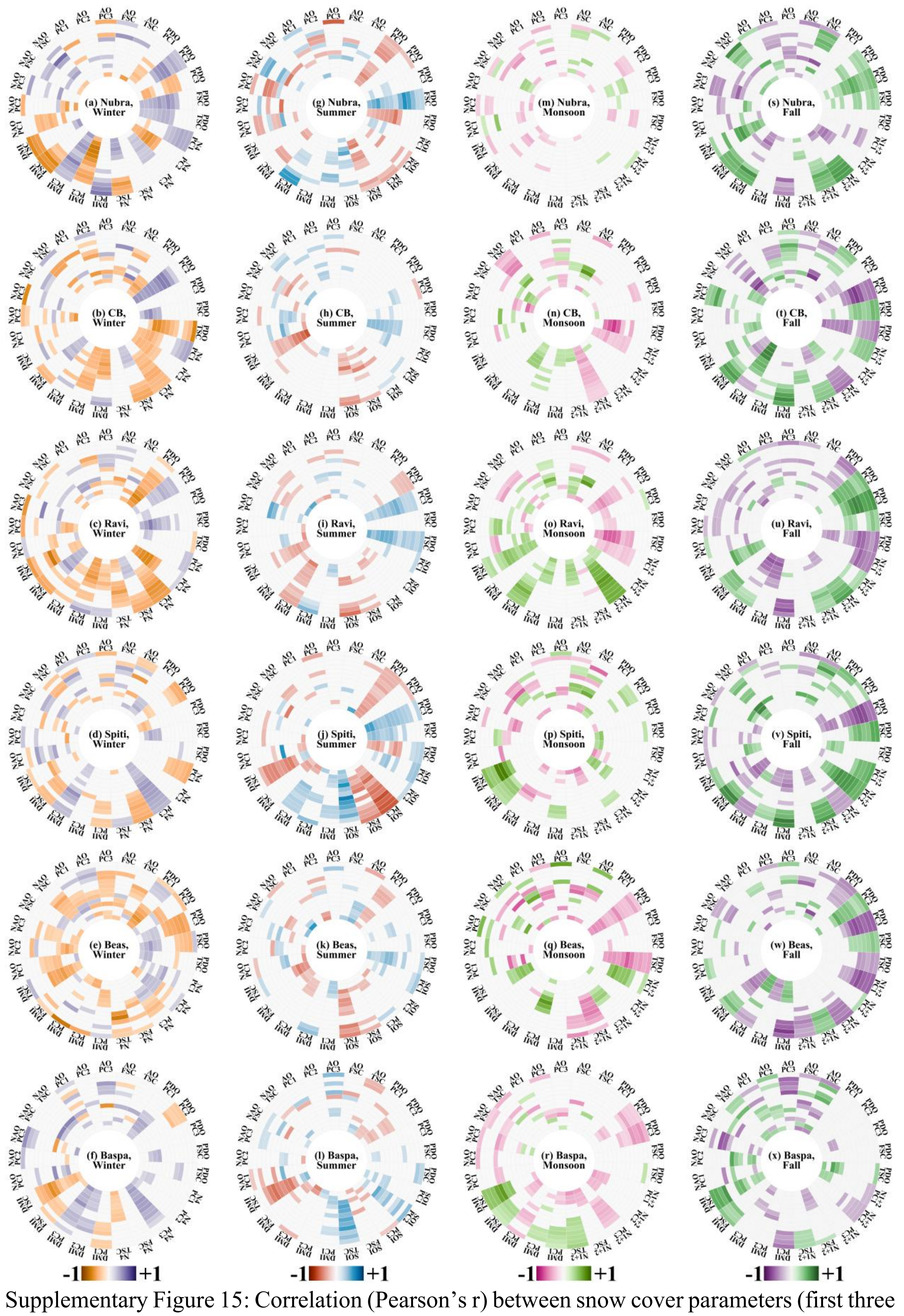

Supplementary Figure 15: Correlation (Pearson's r) between snow cover parameters (first three principal components of snow cover, fractional snow cover, and fractional temporary snow cover) and potential drivers (oceanic-atmospheric indices) - during periods of DMI outside of

its 1standard deviation range. In all the plots, the circle closest to center represents the correlation at lag 0. Concentric circles outwards stand for increasing lags of oceanic-atmospheric indices, with the final perimeter circle standing for lag 12. Each circle is divided into 25 sectors – combinations of five oceanic-atmospheric indices and the five snow cover parameters that were tested for correlation. These combinations are denoted outside the perimeter. For winter (subplots a-f), the legend runs from orange to purple, denoting -1 to +1. The same range of values are represented with a red to blue legend for summer (subplots g-l). A red to green legend is used to depict the r values obtained for monsoon (subplots m-r). For fall finally (subplots s-x), r values are shown with a purple to green legend.

Supplementary Figure 16: Correlation coefficients between oceanic-atmospheric indices and snow cover parameters, during periods of ENSO indices outside of their 1standard deviation ranges

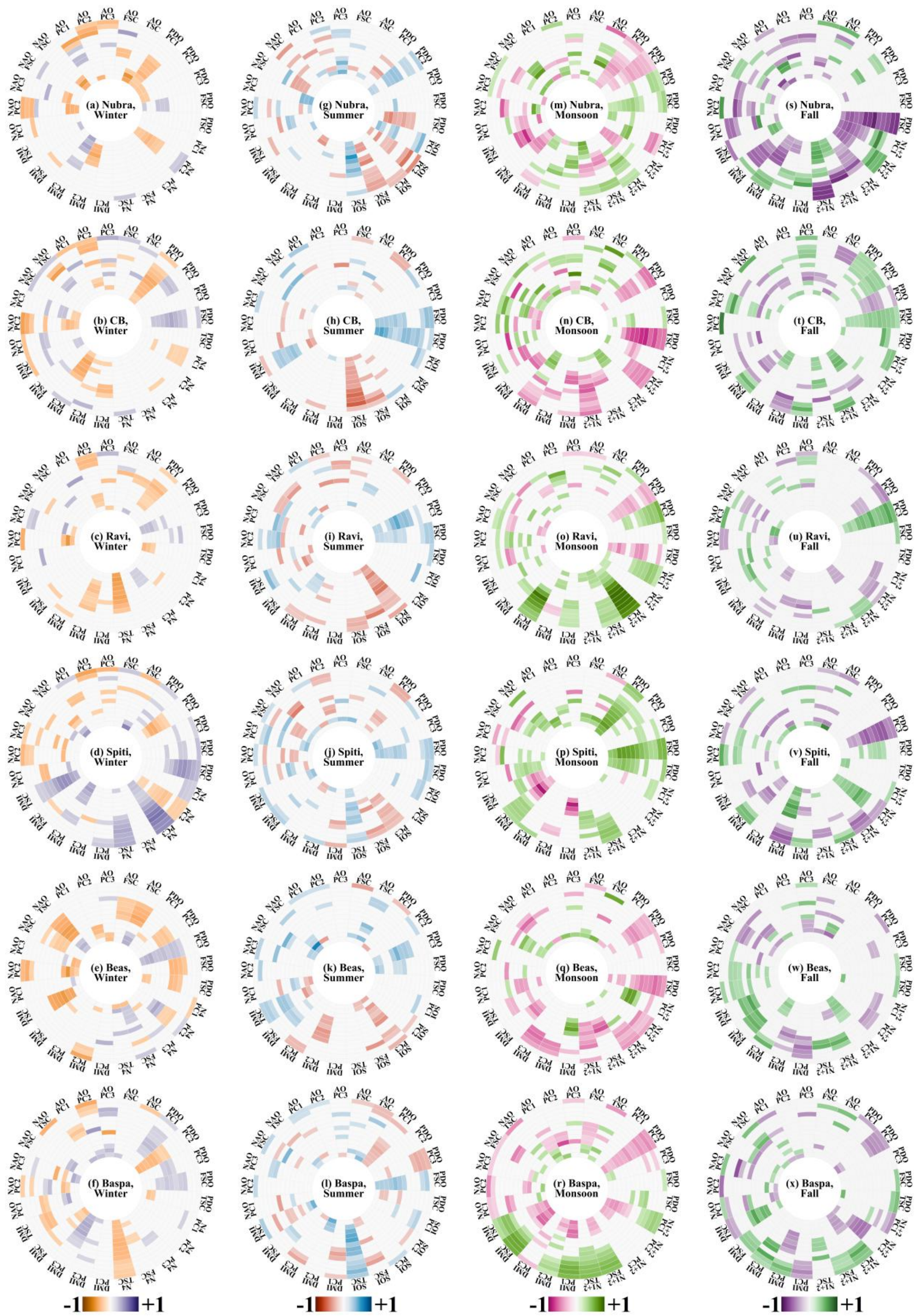

Supplementary Figure 16: Correlation (Pearson's r) between snow cover parameters (first three principal components of snow cover, fractional snow cover, and fractional temporary snow cover) and potential drivers (oceanic-atmospheric indices) - during periods of ENSO indices

outside of their 1standard deviation ranges. In all the plots, the circle closest to center represents the correlation at lag 0. Concentric circles outwards stand for increasing lags of oceanic-atmospheric indices, with the final perimeter circle standing for lag 12. Each circle is divided into 25 sectors – combinations of five oceanic-atmospheric indices and the five snow cover parameters that were tested for correlation. These combinations are denoted outside the perimeter. For winter (subplots a-f), the legend runs from orange to purple, denoting -1 to +1. The same range of values are represented with a red to blue legend for summer (subplots g-l). A red to green legend is used to depict the r values obtained for monsoon (subplots m-r). For fall finally (subplots s-x), r values are shown with a purple to green legend.

Supplementary Figure 17: Lower bounds (for 95 % confidence interval) to estimated regression coefficients from best subset regression.

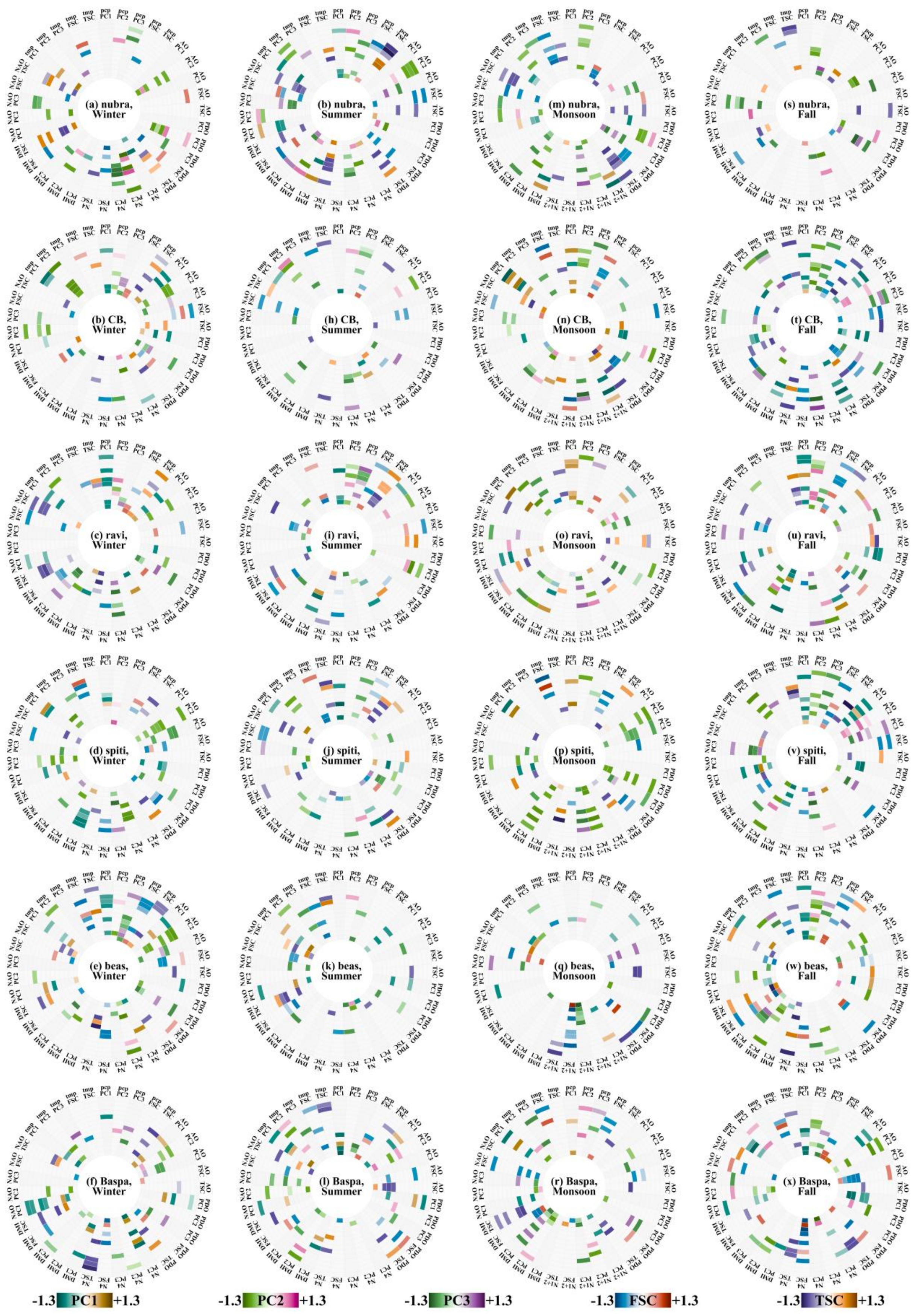

Supplementary Figure 17: Lower bounds to estimated regression coefficients from best subset regression (95% confidence interval). Snow cover parameters (first three principal components, fractional snow cover, and fractional temporary snow cover) and the independent

variable (oceanic-atmospheric index, temperature and precipitation) combination is marked on the perimeter. Concentric circles represent lags of these independent variables, with the centermost circle being lag 0 and the outermost standing for lag 12. The coefficients for PC1 are plotted in a green-brown scale, for PC2 in green-pink, and for PC3 in green-purple. The legends for FSC and TSC are in a blue-red, and orange-purple scale, respectively. The values have been plotted with a contrast stretch of the form $\frac{x}{|x|^{0.8}}$ to ensure that higher values do not overshadow the smaller ones.

Supplementary Figure 18: Upper bounds (for 95 % confidence interval) to estimated regression coefficients from best subset regression.

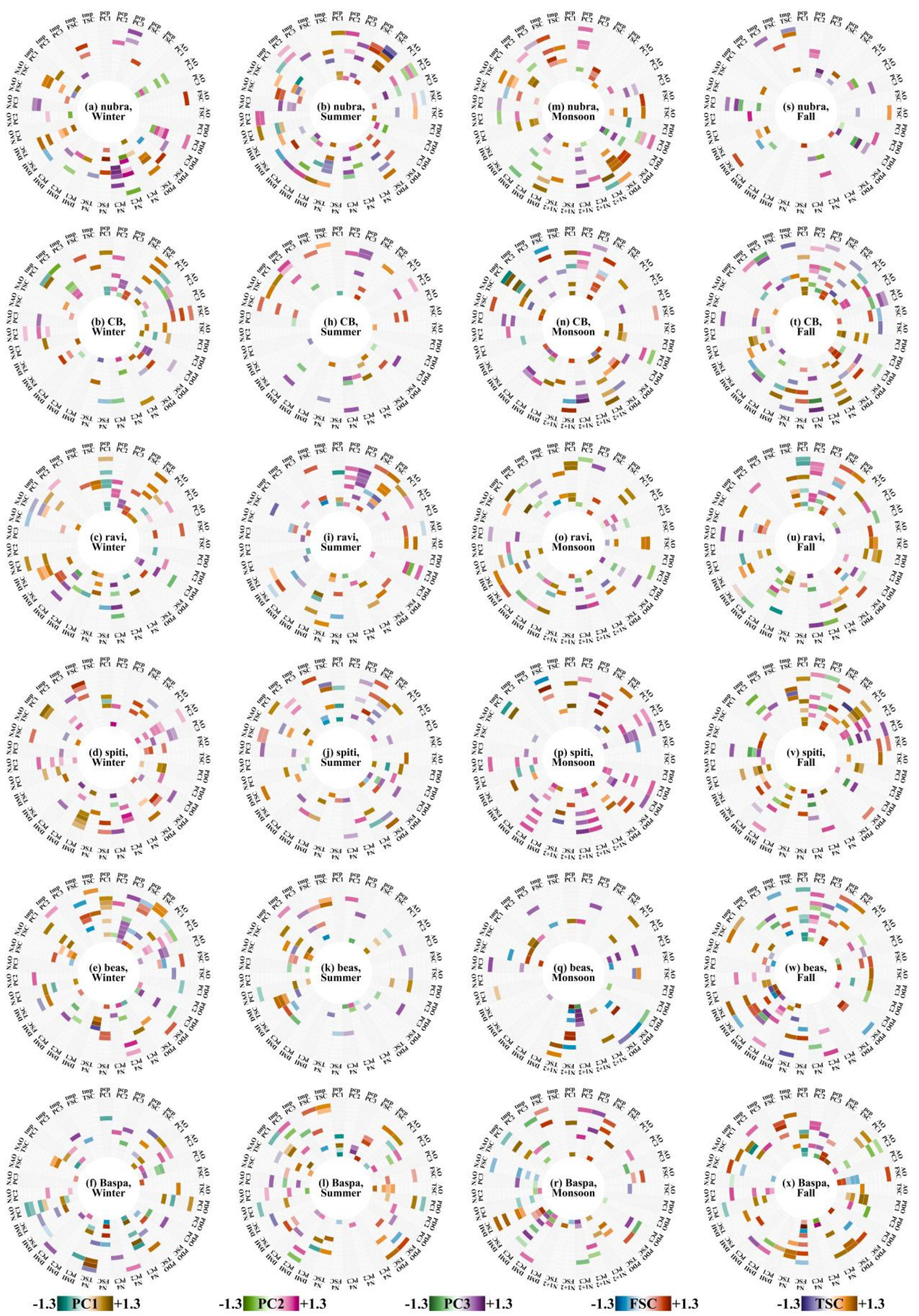

Supplementary Figure 18: Upper bounds to estimated regression coefficients from best subset regression (95% confidence interval). Snow cover parameters (first three principal components, fractional snow cover, and fractional temporary snow cover) and the independent

variable (oceanic-atmospheric index, temperature and precipitation) combination is marked on the perimeter. Concentric circles represent lags of these independent variables, with the centermost circle being lag 0 and the outermost standing for lag 12. Upper bounds to the estimated regression coefficients coefficients for PC1 are plotted in a green-brown scale, for PC2 in green-pink, and for PC3 in green-purple. The legends for FSC and TSC are in a blue-red, and orange-purple scale, respectively. The values have been plotted with a contrast stretch of the form $\frac{x}{|x|^{0.8}}$ to ensure that higher values do not overshadow the smaller ones.

Equations and explanations

1. Normalized Difference Snow Index (NDSI) is calculated as follows:

$$NDSI = \frac{GREEN - SWIR}{GREEN + SWIR} \tag{1}$$

Where Green is reflectance in 0.53µm -0.59 µm wavelength region and SWIR1 is reflectance in the 1.57 µm -1.65 µm window of the electromagnetic spectrum.

2. Fractional Snow Cover (FSC) is given by:

$$Fractional\ snow\ covered\ area\ (FSC) = \frac{Area\ under\ snow\ cover}{Area\ of\ the\ Basin} \tag{2}$$

Area under snow cover is calculated by counting the number of pixels with NDSI values (according to supplemental equation 1) > 0

3. Here the form of the data matrix X used for PCA is given in equation 3

$$X = \begin{bmatrix} Pixel\ 1, Obs\ 1 & \cdots & Pixel\ k, Obs\ 1 \\ \vdots & \ddots & \vdots \\ Pixel\ 1, Obs\ n & \cdots & Pixel\ k, Obs\ n \end{bmatrix} \tag{3}$$

A detailed explanation of Principal Component Analysis can be found in Greenacre et al (2022).

4. Wavelet Coherence requires a mother wavelet (basis function) to transform the original time series (in our case snow cover parameters and oceanic-atmospheric indices) to a time-frequency space. Here the mother wavelet used is a Morlet, described as:

$$\Psi_0(\eta) = \pi^{-1/4}\, e^{i\omega_0\eta} e^{-\eta^2/2} \tag{4}$$

Where η is a non-dimensional time parameter, and $\omega_0$is a non-dimensional frequency parameter. An overview of the wavelet coherence technique can be found in Torrence and Compo (1998). We used a bias corrected cross wavelet transform to compute coherence. Our analysis was conducted with 300 Monte Carlo simulations. Only the significant coherence signals inside the cone of influence were finally interpreted.

5. Pearson's correlation (r) is calculated as follows:

$$r = \frac{n(\sum xy) - (\sum x)(\sum y)}{\sqrt{[n\sum x^2 - (\sum x)^2][n\sum y^2 - (\sum y)^2]}} \quad (5.1)$$

Where n is the number of observations, x and y are observations of the independent (oceanic-atmospheric index) and dependent (snow cover parameter) variables, respectively.
Pearson's correlation (r) is not meant to measure non-linear associations. A test for non-linear correlation can be made through Spearman's ρ, given as

$$\rho = 1 - \frac{6\sum d_i^2}{n(n^2 - 1)} \quad (5.2)$$

Where $d_i$is the difference between the ranks of each observation, and n is the number of observations. Spearman's ρ is actually the Pearson correlation between the rank variables.
A more robust test for non-linear association can be made through Kendall's τ, constructed as:

$$\tau = \frac{number\ of\ concordant\ pairs - number\ of\ discordant\ pairs}{number\ of\ pairs} \quad (5.3)$$

6. Mallow's $C_p$is given as:

$$C_p = k + 1 + \frac{(MSE_k - MSE_{all})(n - k - 1)}{MSE_{all}} \quad (6)$$

Here n is sample size (number of observations), k is the number of drivers (temperature, precipitation, and oceanic atmospheric indices and their lags), and MSE_k is the Mean Square Error of the linear model of the form $snow\ cover\ parameter = constant + coefficient_1 \cdot driver_1 + coefficient_2 \cdot driver_2 + \cdots + coefficient_k \cdot driver_k$. Across all the linear models of a snow cover parameter using different numbers of drivers, the one with the lowest C_p is considered to be the best model. This model explains the most variance with the least variables.

7. Variance explained, or R squared is given as:

$$R^2 = 1 - \frac{Sum\ of\ Squares\ of\ residuals\ (RSS)}{Total\ sum\ of\ Squares\ (TSS)} \quad (7.1)$$

Introducing more and more independent variables can keep increasing the value of R^2. Adjusted R squared addresses this problem by having a penalty associated with including independent variables. Adjusted R squared is calculated as:

$$R_{adj}^2 = 1 - \frac{(1 - R^2)(N - 1)}{(N - p - 1)} \quad (7.2)$$

where N is the sample size (observations), and p is the number of predictors.

8. Normalization of a variable (in our case climate variability mode indices, temperature, precipitation, and the five snow cover attributes) is performed as:

$$v_i = \frac{v_i - v_{mean}}{v_{max} - v_{min}} \quad (8)$$

Where v_i is the ith observation of a variable, v_{mean} is the arithmetic mean taken over all observations of v, v_{max} is the maximum observed value of v, and v_{min} is the minimum observed value of v.

Tables

Supplementary Table 1: Variance explained by first three PCs

Basin	Time period	PC1	PC2	PC3
Baspa	Summer	0.5276	0.6851	0.73958
	Monsoon	0.6771	0.76585	0.80299
	Fall	0.5858	0.7038	0.7434
	Winter	0.5381	0.6788	0.74900
	All time	0.6308	0.76680	0.810460
Beas	Summer	0.436	0.6153	0.69428
	Monsoon	0.5874	0.6971	0.73361
	Fall	0.4347	0.5697	0.65641
	Winter	0.4306	0.6164	0.7236
	All time	0.5185	0.6410	0.72505
ChandraBhaga	Summer	0.4813	0.6129	0.66619
	Monsoon	0.6006	0.6803	0.72505
	Fall	0.5313	0.61789	0.67261
	Winter	0.5343	0.6382	0.71137
	All time	0.6188	0.7331	0.76926
Nubra	Summer	0.3503	0.4916	0.54372
	Monsoon	0.4838	0.55600	0.61018
	Fall	0.3708	0.4733	0.53839
	Winter	0.3019	0.4951	0.56775
	All time	0.4589	0.6076	0.65981
Ravi	Summer	0.4297	0.5709	0.64672
	Monsoon	0.4623	0.54576	0.61514
	Fall	0.3952	0.5540	0.62635
	Winter	0.4821	0.6559	0.74071
	All time	0.5222	0.6336	0.71343
Spiti	Summer	0.496	0.1267	0.06106
	Monsoon	0.5146	0.6330	0.69023
	Fall	0.4674	0.5732	0.65891
	Winter	0.5156	0.61415	0.68828
	All time	0.55	0.653	0.72046

Supplementary Table 1: Cumulative Variance explained by the first three PCs, for each basin, each season, and the full observation period.

Supplementary Table 2: R^2_{adj} of models derived from best subset regression.

	Snow Cover Parameter	Winter	Summer	Monsoon	Fall
Baspa	PC1	0.730	0.716	0.430	0.630
	PC2	0.152	0.404	0.457	0.528
	PC3	0.389	0.160	0.397	0.248
	FSC	0.342	0.603	0.588	0.717
	TSC	0.415	0.394	0.229	0.320

Beas	PC1	0.602	0.586	0.608	0.727
	PC2	0.332	0.140	0.122	0.404
	PC3	0.297	0.393	0.504	0.468
	FSC	0.528	0.638	0.816	0.783
	TSC	0.520	0.585	0.523	0.663
ChandraBhaga	PC1	0.509	0.224	0.719	0.403
	PC2	0.536	0.281	0.233	0.373
	PC3	0.447	0.267	0.200	0.748
	FSC	0.430	0.153	0.626	0.404
	TSC	0.417	0.369	0.590	0.684
Nubra	PC1	0.457	0.609	0.548	
	PC2	0.673	0.551	0.591	0.482
	PC3	0.217	0.367	0.405	0.474
	FSC	0.277	0.291	0.352	0.192
	TSC	0.592	0.860	0.456	0.534
Ravi	PC1	0.629	0.410	0.624	0.718
	PC2	0.388	0.170	0.510	0.683
	PC3	0.404	0.357	0.574	0.397
	FSC	0.453	0.750	0.472	0.585
	TSC	0.587	0.696	0.634	0.374
Spiti	PC1	0.476	0.735	0.658	0.602
	PC2	0.324	0.145		0.636
	PC3	0.223	0.306	0.301	0.732
	FSC	0.252	0.723	0.565	0.303
	TSC	0.380	0.472	0.730	0.485

Supplementary Table 2: R^2_{adj} (as given in in supplemental equation 7.2) for the models selected through best subset regression. The best model is chosen using Mallow's $C_p$as described in supplemental equation 7.

Additional text

Supplementary Text 1: Interpretation of composite analysis

We found that in winters of enhanced AO activity (Supplementary Figures 12(a-f)), the lag between AO and PC3 reduces to 0-3 months from the higher lags of 6-10 months noted previously. The higher lag connections also remain, possibly due to persistence in the climatic state of the arctic, which starts building up and affecting snow cover since fall (Supplementary Figures 12(s-x)). Further, we also see an enhanced association between DMI and PC1/PC2 (depending on the basin), at lower lags (Supplementary Figures 12(a-f). A similar effect is seen on the DMI-PC2/PC3 association during winters of stronger NAO states (Supplementary Figures 13(a-f). Furthermore, during winters of enhanced DMI activity (Supplementary Figures 15(a-f)), strong associations between various aspects of snow cover and both NAO and AO were consistently observed. Given that we had not found any major effects of DMI on winter snow cover in the correlation analysis, the strengthening of AO/NAO – snow cover associations observed during higher DMI activity winters suggest that the correlation between snow cover and DMI at low lags possibly originate from climatic conditions of the wintertime Indian Ocean modulating AO/NAO driven weather systems. Finally, while winters of heightened PDO activity (Supplementary Figures 16(a-f)) do not show consistent concurrent intensification of association between any other climate variability modes and their effects on

snow cover, times of higher ENSO activity (Supplementary Figure 16(a-f)) appear to be synchronous with higher PDO-snow cover associations.

For summers with enhanced AO activity (Supplementary Figures 12(g-l)), AO appears to influence PC1 with a lag of 4-6 months. This stronger association between AO and PC1 at higher (longer than 1 season) lag possibly is only a stronger carry-over effect from winter, a time when snow cover has already been shown to be affected by AO. In fact, summers with strong AO activity are likely to result from stronger AO activity during the previous winter. Moreover, during summers of heightened NAO activity, the link previously noted between NAO and PC1 is strengthened, and the lag reduces from 5 months to 1-3 months across the basins (Supplementary Figures 13(g-l)). This reduction in lag time possibly originates from persistent climatic conditions in the North Atlantic region. Further, heightened AO (Supplementary Figures 12(g-l)) and NAO (Supplementary Figures 13(g-l)) activity appears to coincide with enhanced PDO-snow cover associations. However, summers of heightened PDO activity (Supplementary Figures 14(g-l)) do not show as consistent an association with enhanced AO and NAO activity, suggesting that AO and NAO driven weather systems can regulate the snow cover driving mechanism of PDO. Note that the AO/NAO-DMI (Supplementary Figures 12(g-l)/ Supplementary Figures 13(g-l) – Supplementary Figures15(g-l)) link observed in winter still holds in summer, suggesting that NAO and AO driven weather systems might regulate PDO while being regulated themselves by the Indian Ocean. Finally, unlike in enhanced PDO winters, for summers of similar PDO states (Supplementary Figures 14(g-l)), a clear enhanced ENSO-snow cover association is observed. However, since enhanced ENSO summers (Supplementary Figures 16(g-l)) show a similarly strong PDO-snow cover association, this concurrence might be only an artefact on PDO and ENSO states synchronizing, and not any modulation of snow cover driving mechanisms.

Finally, the composite analysis for monsoon and fall did not yield any new relationships, but only validated the associations determined in the correlation analysis.
